# Diurnal Regulation of SOS Pathway and Sodium Excretion Underlying Salinity Tolerance of *Vigna marina*

**DOI:** 10.1111/pce.15402

**Published:** 2025-01-24

**Authors:** Yusaku Noda, Fanmiao Wang, Sompong Chankaew, Hirotaka Ariga, Chiaki Muto, Yurie Iki, Haruko Ohashi, Yu Takahashi, Hiroaki Sakai, Kohtaro Iseki, Eri Ogiso‐Tanaka, Nobuo Suzui, Yong‐Gen Yin, Yuta Miyoshi, Kazuyuki Enomoto, Naoki Kawachi, Prakit Somta, Jun Furukawa, Norihiko Tomooka, Ken Naito

**Affiliations:** ^1^ Takasaki Institute for Advanced Quantum Science National Institutes for Quantum Science and Technology (QST) Gunma Japan; ^2^ Research Center of Genetic Resources National Agriculture and Food Research Organization Ibaraki Japan; ^3^ Department of Agronomy Faculty of Agriculture Khon Kaen University Khon Kaen Thailand; ^4^ Department of Agronomy Faculty of Agriculture at Kamphaeng Saen Kasetsart University Nakhon Pathom Thailand; ^5^ Graduate School of Frontier Sciences The University of Tokyo Chiba Japan; ^6^ Research Center of Advanced Analysis National Agriculture and Food Research Organization Ibaraki Japan; ^7^ Japan International Research Center for Agricultural Sciences Ibaraki Japan; ^8^ Center for Molecular Biodiversity Research National Museum of Nature & Science Ibaraki Japan; ^9^ Institute of Life and Environmental Sciences University of Tsukuba Ibaraki Japan

**Keywords:** positron‐emitting tracer imaging system, QTL analysis, RNA‐seq, salt tolerance, sodium‐22, *Vigna marina*

## Abstract

*Vigna marina* (Barm.) Merr. is adapted to tropical marine beaches and has an outstanding tolerance to salt stress. Given there are growing demands for cultivating crops in saline soil or with saline water, it is important to understand how halophytic species are adapted to the saline environments. Here we revealed by positron‐emitting tracer imaging system (PETIS) that *V. marina* actively excretes sodium from the root during the light period but not the dark period. The following whole genome sequencing accompanied with forward genetic study identified a QTL region harbouring *SOS1*, encoding plasma membrane Na^+^/H^+^ antiporter, which was associated with not only salt tolerance but also the ability of sodium excretion. We also found the QTL region contained a large structural rearrangement that suppressed recombination across ~14 Mbp, fixing multiple gene loci potentially involved in salt tolerance. RNA‐seq and promoter analyses revealed *SOS1* in *V. marina* was highly expressed even without salt stress and its promoter shared common *cis*‐regulatory motifs with those exhibiting similar expression profiles. Interestingly, the *cis‐*regulatory motifs seemed installed by a transposable element (TE) insertion. Though not identified by genetic analysis, the transcriptome data also revealed *SOS2* transcription was under diurnal regulation, explaining the pattern of sodium excretion together with upregulated expression of *SOS1*. Altogether, the study elucidated one aspect of the strategy adopted by *V. marina* to adapt to marine beach, which is highly saline and transpiring.

## Summary statement

We found diurnal regulation of SOS pathway underlies the extraordinary salinity tolerance of *Vigna marina*. In *V. marina*, SOS1 is highly expressed, but SOS2 expression is high in daytime and low at night, leading to the diurnal regulation of Na^+^ excretion.

## 1 Introduction

*Vigna marina* (Barm.) Merr. and *Vigna luteola* (Jacq.) Benth. are the most and the second most salt‐tolerant species in the genus *Vigna* (Iseki et al. 2016). They are oceanic dispersal species and dominate the vegetation of tropical and subtropical beaches (Tomooka 2002). Given the increasing problems of soil salinization and freshwater shortage, it is important to understand its mechanisms of salt tolerance and the underlying genetics for developing practical salt‐tolerant crops.

The preceding studies have revealed the outstanding performance of *V. marina* under salt stress. It survives 500 mM NaCl for more than 8 weeks (Yoshida et al. 2020), maintains root growth in 200 mM NaCl (Wang et al. 2024), develops thicker apoplastic barrier in response to salt stress (Wang et al. 2025) and shows higher photosynthetic rate and stomatal conductance in 150 mM NaCl than in control (Yoshida et al. 2020). While some accessions of *V. luteola* also showed remarkable performance up to 350 mM NaCl, others originally collected from riverbanks did not tolerate the condition of 150 mM NaCl (Chankaew et al. 2014; Yoshida et al. 2020; Wang et al. 2024). However, the sensitive accessions of *V. luteola* are still more salt‐tolerant compared to *Vigna angularis* (Willd.) Ohwi and Ohashi, which is azuki bean, one of the major *Vigna* crops (Iseki et al. 2016). The following study of ours, by tracer experiment with radioactive sodium (^22^Na), revealed *V. marina* allocates the least amount of sodium in leaves, stems and roots compared to other species (Noda et al. 2022). Thus, the ability of sodium exclusion underlies its extraordinary tolerance to salt stress.

However, still unknown is whether *V. marina* simply suppresses sodium uptake from the root, or actively excretes sodium that is once loaded to xylem. Moreover, genetic factors responsible for sodium exclusion must be identified to fully understand how *V. marina* is adapted to saline environments such as marine beaches.

**Figure 1:**
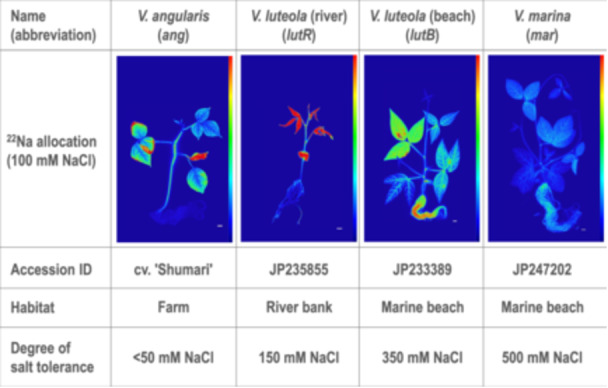
Sodium allocation and degree of salt tolerance in the plant materials. Sodium allocation was visualized with autoradiography using ^22^Na. Colour scales indicate the relative intensity of radioactivity, where blue and red correspond to low and high, respectively.

Thus, in this study, we adopted the positron‐emitting tracer imaging system (PETIS) (Uchida et al. 2004; Fujimaki et al. 2015), which enables real‐time mapping of sodium in living plants of *V. marina* and other accessions with various degrees of salt tolerance (Figure 1) (Yoshida et al. 2016; Chankaew et al. 2014; Yoshida et al. 2020). Then we performed a forward‐genetic approach by crossing *V. marina* and *V. luteola* to identify genetic loci involved in salt tolerance. We also reconstructed a new reference genome of *V. marina* and *V. luteola* and performed some comparative genomic/transcriptomic studies to identify the responsible genes and genetic variations underlying the differential expressions. The knowledge obtained in this study will provide insights regarding how to use the known genes to make a plant adapted to saline environments.

## 2 Materials and Methods

### 2.1 Plant Materials and Growth Condition

All plant seeds were provided by the NARO Genebank in Tsukuba, Japan (https://www.gene.affrc.go.jp/index_en.php) (Figure 1). Seeds were sterilized by shaking in 70% ethanol for 5 min and then shaking another 5 min in 0.5% sodium hypochlorite. The sterilized seeds were germinated on Seramis clay (Westland Deutschland GmbH, Mogendorf, Germany) for 1 week and then transferred to hydroponic solution in a growth chamber (light: 28°C for 14 h, dark: 25°C for 10 h, light intensity 500 µM^−1^ s^−1^ m^−2^). Each plant was cultivated for the following number of days: *V. angularis* 10 days, *V. luteola* (river) 14 days, *V. luteola* (beach) 14 days, *V. marina* 17 days. The hydroponic solution contained a diluted nutrient solution of OAT House No.1 (1.5 g L^−1^): OAT House No.2 (1 g L^−^^1^) (Otsuka Chemical Co, Japan) in a 1:1 ratio with concentrations of 18.6 mEq L^−1^ nitrogen, 5.1 mEq L^−1^ phosphorus, 8.6 mEq L^−1^ potassium, 8.2 mEq L^−1^ calcium and 3.0 mEq L^−1^ magnesium.

### 2.2 Visualization of ^22^Na Allocation

To visualize ^22^Na allocation, pre‐cultured plants were transferred to the Center for Research in Radiation, Isotopes and Earth System Sciences at the University of Tsukuba, Japan. The plants were transplanted into a new hydroponic solution containing 5 kBq ^22^Na (PerkinElmer, USA) with non‐radioactive 100 mM ^23^NaCl. After adding the radioisotope, the plants were again incubated under long‐day conditions (light: 28°C for 14 h, dark: 25°C for 10 h, light intensity 200 µmol s^−1^ m^−2^) for 3 days. After incubation, the roots were carefully washed and the whole plant body was placed in a plastic bag and exposed to a storage phosphor screen (BAS‐IP‐MS‐2025E, GE Healthcare, UK) in Amersham exposure cassettes (GE Healthcare, UK) for 24 h. The exposed screen was then scanned with a laser imaging scanner Typhoon FLA‐9500 (GE Healthcare, UK). All experiments were performed independently on more than three biological replicates. To arrange radioactive intensity equally at each image, photo‐stimulated luminescence and contrast were equalized by Multi Gauge version 3.0 (Fujifilm, Japan). In an experiment to visualize ^22^Na allocation in *V. marina* under different salt stresses, plants were transplanted into 100, 200 and 300 mM ^23^NaCl hydroponic solutions containing 5 kBq ^22^Na. Subsequent procedures were as described above.

### 2.3 PETIS Imaging and Analysis

The visualization of sodium excretion from plant roots was carried out using PETIS at the Takasaki Institute for Advanced Quantum Science, Japan. Initially, a polytetrafluoroethylene sheet with holes was placed in an 8 cm high acrylic cell as a partition to enable quantification of ^22^Na radioactivity from hydroponic culture. A silicone tube was then installed for injecting the ^22^Na solution into the acrylic cell, which was attached to an acrylic board. The plants of 2 weeks old, were inserted into the acrylic cell and stabilized with a black urethane sponge. The plants were transferred to a growth chamber equipped with the PETIS detector. Prior to treatment with ^22^Na, the plants were pretreated with a hydroponic solution containing 100 mM of non‐radioactive NaCl for 1 h. After 1 h, the hydroponic solution was exchanged with a fresh solution containing 100 mM of non‐radioactive NaCl and 500 kBq ^22^Na to feed the plants for 24 h. Then, the ^22^NaCl hydroponic solution was exchanged twice with ice‐cold 10 mM CaCl_2_ solution to wash the roots. After washing, the plants were transplanted to 100 mM NaCl hydroponic solution without ^22^Na and the data collection by PETIS was started. During PETIS imaging, the surface of the hydroponic solution was maintained by a siphon pump, and roots were shaded with aluminium foil. Growth conditions during imaging were as follows: Light: 28°C for 14 h, dark: 25°C for 10 h, light intensity: 200 μmol s^−1^ m^−2^.

PETIS collected data every minute for 48 or 72 h. The original images acquired every minute were then reintegrated into montage images at 1 h intervals. Quantification of ^22^Na in the hydroponic solution, root and shoot was done by defining a region of interest (ROI) with ImageJ (National Institutes of Health, Bethesda, MD, USA; http://rsb.info.nih.gov/ij/). Time‐course analysis of ^22^Na radioactivity within each ROI was performed by generating a time‐activity curve (TAC) from signal intensities using ImageJ.

All the experiments were independently performed more than three times with three or four biological replicates.

### 2.4 Genome Sequencing and Assembly

As described by Naito (2023), we extracted genomic DNA from the unexpanded leaves with Nucleobond HMW DNA kit (MACHEREY‐NAGEL GmbH & Co. KG, Düren, Germany), size‐selected the DNA with Short Read Eliminator XL kit (Pacific Biosciences of California Inc., California, USA), and prepared three libraries for nanopore sequencing with SQK‐LSK109 (Oxford Nanopore Technologies KK, Tokyo, Japan). Each library was then loaded to MinION R9.4.1 Flowcell (Oxford Nanopore Technologies KK, Tokyo, Japan) and was run for 72 h. The obtained raw data was transformed into fastq format with bonito‐0.2.1. We also obtained short reads with HiSeq 4000 (Illumina, San Diego, USA), which was provided as a customer service by GeneBay Inc. (Yokohama, Japan).

For draft assembly, we used necat‐0.0.1 (Chen et al. 2021) with default parameters except ‘PREP_OUTPUT_COVERAGE = 60’ and ‘CNS_OUTPUT_COVERAGE = 40’. The contigs were polished twice with racon‐1.4.3 (Vaser et al. 2017) and once with medaka‐1.0.3 (https://github.com/nanoporetech/medaka) using long reads. We also performed further polishing with Hypo‐1.0.3 (Ritu, Joshua, and Wing‐Kin 2019) using short reads.

The polished contigs were then scaffolded into pseudomolecules by anchoring to the linkage map (see below). When we found controversies between contigs and the linkage map, we manually fixed the misassemblies as described by Sakai et al. (2015). The pseudomolecules of *V. marina* were then annotated with ORFs exactly as described in our previous study (Naito et al. 2022). Those of *V. luteola* were annotated by lifting those of *V. marina* with GeMoMa‐1.9 (Keilwagen et al. 2018), as described in another study of ours (Ito et al. 2024). We also annotated transposable elements (TEs) using EDTA‐1.9.6 (Ou et al. 2019) with default parameters.

### 2.5 QTL Analysis

We crossed *V. marina* and *V. luteola* (beach), selfed the F_1_ plant, and obtained F_2_ seeds. To generate replicates, the F_2_ plants were cultivated in hydroponic culture as described in Y. Yoshida et al. (2016) for 8 weeks and were clonally propagated by cutting (Supporting Information S1: Figure 1). For phenotyping, three clones of each F_2_ plant were cultivated in hydroponic culture with 50 mM NaCl for a week, transferred to the new culture containing 350 mM NaCl for 8 weeks, and then evaluated for three traits, ‘wilt score’, ‘shoot generation score’ and ‘stay green score’. For the wilt score, the F_2_ clones were scored as 1, 3, 5, 7 or 9 according to the salt damage as shown in Supporting Information S1: Figure 1. For the shoot generation score, the clones were scored as 3 if they kept growing and generating new branches or 1 if otherwise. For the stay green score, the clones were scored as 3 if all the leaves stayed green during the experiment or 1 if otherwise. For genotyping, genomic DNA was extracted from the young leaves of each F_2_ plant with the CTAB method. The extracted DNA was then used for preparing restriction site‐associated DNA sequencing (RAD‐seq) (Baird et al. 2008) libraries as described by Matsumura et al. (2014), except *Bam*HI and *Mbo*I were used for DNA digestion. The prepared library was sequenced with HiSeq 2000 (Illumina), which was provided as a customer service by GeneBay Inc. (Yokohama, Japan). The obtained sequence data were mapped to the *V. marina* genome with bwa (Li and Durbin 2010) and then processed with Stacks‐1.19 (Catchen et al. 2013) to obtain genotypes. The obtained genotype data was then analysed for linkage with onemap (Taniguti et al. 2023) to construct a linkage map, which was also used for scaffolding contigs (see above). The association between the phenotypes and the genotypes was then estimated with R/qtl2 (Broman et al. 2019).

### 2.6 Transcriptome Analysis

#### 2.6.1 1st Experiment

Before initiation of salt stress, plants of *V. marina* were grown in hydroponic culture for 2 weeks and pre‐treated with 50 mM NaCl for 3 days. Then, at 11 am, we transferred the plants to hydroponic culture containing 200 mM NaCl. After the initiation of salt stress, we collected leaves and roots of three plants at 24, 36, 48 and 60 h. The collected leaves and roots were immediately wrapped with foil and frozen in liquid nitrogen. From the collected samples, total RNA was extracted with RNeasy Plant Mini Kit (Qiagen KK, Tokyo, Japan). From the extracted RNA, we prepared libraries for 3'mRNA‐seq with Collibri 3'mRNA Library Preparation Kit for Illumina Systems (Thermo Fisher Scientific K.K., Tokyo, Japan). The prepared libraries were sequenced with HiSeq 4000 as a customer service of GeneBay Inc. (Yokohama, Japan). After sequencing, we estimated the read counts of each gene of *V. marina* by kallisto‐0.45.2 (Bray et al. 2016). With edgeR (Chen, Lun, and Smyth 2016), we separately analysed the leaf data set and the root data set. We first normalized the read counts and extracted differentially expressed genes (DEGs), which were not only significant as ANOVA but also at least one of the data points had twofold or greater difference compared to the average. The extracted gene sets were then clustered with SOM‐clustering (Wehrens and Buydens 2007).

#### 2.6.2 2nd Experiment

*V. angularis, V. luteola* (river), *V. luteola* (beach) and *V. marina* were grown, salt‐stressed, sampled and sequenced as described above. The RNA‐seq data was quantified using the quasi‐mapping‐based mode of Salmon (Patro et al. 2017) with indexing of a set of primary transcripts of *V. marina*. The resulting raw counts were TMM (Trimmed mean of M values)‐normalized using edgeR (Chen, Lun, and Smyth 2016) and the resulting TMM‐normalized values were further clustered using SOM (Wehrens and Buydens 2007) with a map size of 8 × 8.

### 2.7 Promoter Analysis

All the promoter analysis was done with MEME Suite (Bailey et al. 2015). First, we extracted the insertion sequence found in the upstream of *VmSOS1* and scanned it with meme command (Bailey and Elkan 1994) with an option of ‘‐m mod anr’. The output was screened for Arabidopsis DAP motifs (O'Malley et al. 2016) using tomtom command (Gupta et al. 2007) with default settings.

For motif scanning, we used fimo command (Grant, Bailey, and Noble 2011) to screen the promoters of the selected gene sets for the Arabidopsis DAP motifs and counted the numbers of ‘ERF1’ and ‘CRF10’ from the output. We used binominal distribution test to compare the probability between the selected gene sets versus all genes as control.

## 3 Results

### 3.1 ^22^Na‐Allocation in *V. marina* and Its Relatives

In addition to our previous study that revealed the ^22^Na allocation in *V. marina, V. luteola* (beach) and *V. angularis* (Noda et al. 2022), we visualized that in *V. luteola* (river) in the condition of 100 mM NaCl (Figure 1). The result of *V. luteola* (river) showed a typical pattern of glycophytes, which was similar to *V. angularis*, presenting high ^22^Na allocation to leaves and low allocation to roots. In contrast, *V. luteola* (beach) allocated more ^22^Na to roots and less to leaves and stems, while *V. marina* highly restricted ^22^Na allocation even in roots.

### 3.2 Sodium Excretion and Culture Alkalization by *V. marina*

**Figure 2:**
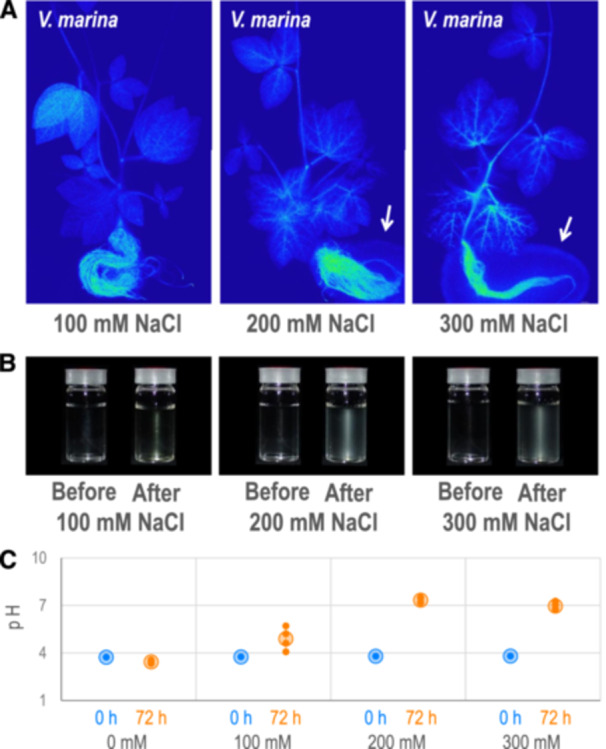
Sodium excretion and culture alkalization by *Vigna marina*. (A) Autoradiographs of *V. marina* plants fed with 100, 200 or 300 mM NaCl containing ^22^Na. White arrows indicate traces of exudated ^22^Na from the root. (B) Hydroponic culture solution before and after cultivation of *V. marina*. (C) pH of hydroponic culture solution before (0 h) and after (72 h) cultivation of *V. marina*. Small dots indicate values of biological replicates, while larger plots and error bars indicate mean ± SD, respectively. [Color figure can be viewed at wileyonlinelibrary.com]

To elucidate to which intensity of salt stress *V. marina* is able to keep sodium out of the plant, we fed the plants with ^22^Na in conditions of 100, 200 and 300 mM NaCl (Figure 2A). The results revealed that *V. marina* kept sodium allocation low even under 300 mM NaCl condition. Instead, we found clear traces of ^22^Na around the root of the plants in 200 mM NaCl and more in 300 mM NaCl.

During the experiment, we noticed that the hydroponic culture containing NaCl turned cloudy after plant cultivation for 72 h (Figure 2B, Supporting Information S1: Figure 2). The culture with 100 mM NaCl formed a little precipitation, while that of 200 or 300 mM NaCl formed more precipitation.

As we suspected that the precipitation was the nutrient salts because the activity of Na^+^/H^+^ antiporters could alkalize the hydroponic culture by depleting proton, we measured the pH of the hydroponic culture before and after cultivating the plants of *V. marina* (Figure 2C). As a result, with 0 mM NaCl, the plant cultivation declined the culture pH from 3.7 to 3.4, while it increased to 4.9 in 100 mM NaCl, 7.3 in 200 mM NaCl and 6.9 in 300 mM NaCl.

### 3.3 Dynamics of ^22^Na in a Living Plant Body of *V. marina*

**Figure 3:**
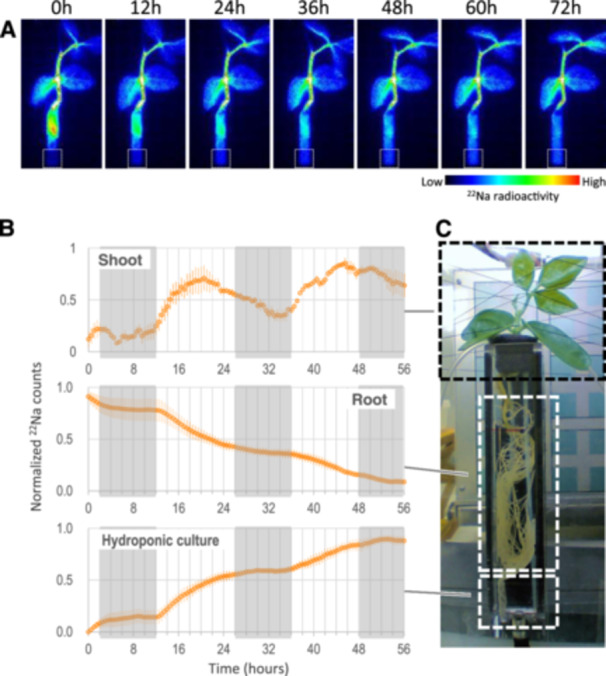
Dynamics of sodium transport in *Vigna marina*. (A) Time‐lapse images of gamma irradiation mapped by PETIS. A plant of *V. marina* was fed with ^22^Na for 24 h and then time‐lapsed. White boxes indicate the area of hydroponic culture measured for ^22^Na. (B) Indexed amount of irradiation across time in the shoot, the root and the hydroponic culture media. Light and dark periods are indicated as white and grey in the plot, respectively. Error bars indicate standard errors. (C) Setup of PETIS. [Color figure can be viewed at wileyonlinelibrary.com]

To observe sodium dynamics in a plant of *V. marina*, we performed real‐time mapping of ^22^Na using PETIS (Figure 3A, Supporting Information S1: Figure 3 and Video). The real‐time images revealed that, with time, the ^22^Na decreased in the root while increased in the hydroponic culture. From the images, we did not notice that the amount of ^22^Na in the shoot changed during the experiment.

However, the following quantitative analysis revealed there was a diurnal rhythm in sodium dynamics in *V. marina*. In the shoot, the amount of ^22^Na increased during the light period and decreased during the dark period (Figure 3B), while that in the root kept simply decreasing but faster in the light period and slower in the dark period (Figure 3B). In contrast to the root, the amount of ^22^Na in the hydroponic culture increased faster during the light period and slower or even stopped during the dark period (Figure 3B). Notably, the rate of ^22^Na increase and in the hydroponic culture was maximized in the first 6 h of the light period, indicating *V. marina* actively excreted sodium against the direction of water uptake.

### 3.4 Whole Genome Sequence of *V. marina* and *V. luteola* (Beach)

**Figure 4:**
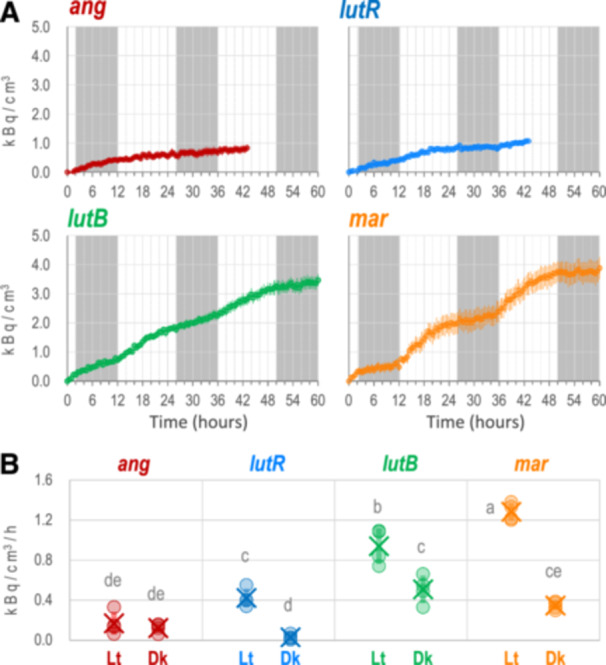
Dynamics of sodium excretion by the four accessions. (A) Concentration of ^22^Na in the culture media across time. *X*‐ and *Y*‐axes indicate time and the estimated concentration of ^22^Na in the hydroponic culture. Light and dark periods are indicated as white and grey in the plot, respectively. Error bars indicate standard errors. (B) Velocity of ^22^Na excretion in the light period (Lt) and the dark period (Dk). The light and the dark period correspond to 12−26 h and 26−36 h, respectively. Different alphabets above the plots indicate significant differences by Tukey−Kramer test (*p* < 0.05). [Color figure can be viewed at wileyonlinelibrary.com]

To facilitate genetic and genomic analyses in this study, we sequenced and assembled the whole genomes of *V. marina* and *V. luteola* (beach) using Oxford Nanopore sequencer (Supporting Information S1: Table 1 and Figure 4A). The draft assembly of *V. marina* comprised 551.5 Mbp in 72 contigs with N50 length of 20.7 Mbp, whereas that of *V. luteola* (beach) was 515.7 Mbp in 146 contigs with N50 length of 18.8 Mbp. We also crossed the two accessions, obtained 286 F_2_ plants that were genotyped by RAD‐seq (Supporting Information S1: Data). With the 286 marker loci obtained, we constructed a high‐density genetic map with 11 linkage groups, which corresponded to the karyotype of most Vigna species (2n = 22) (Supporting Information S1: Figure 4B). We then anchored the draft assemblies to the genetic map and reconstructed the 11 pseudomolecules with high‐quality gene annotation covering more than 98% of BUSCO genes in the Fabaceae family (Supporting Information S1: Figure 4A). The genomes of the two accessions were almost colinear to each other, except inversions on chr1, chr4 and chr7 (Supporting Information S1: Figure 4C).

### 3.5 Diurnally‐Regulated Genes in *V. marina*

**Figure 5:**
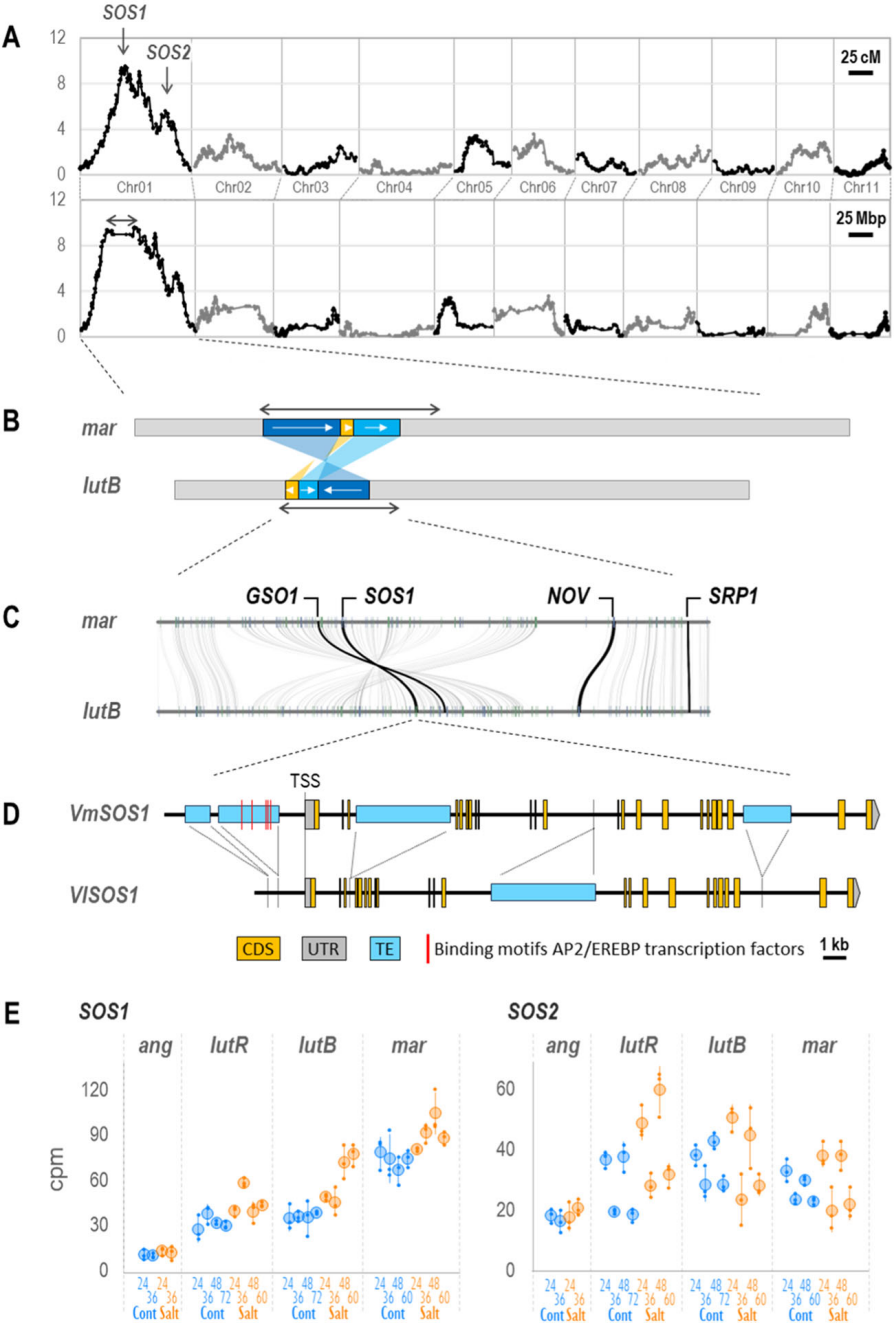
Genetic factors underlying difference of salt tolerance between *Vigna marina* and *Vigna luteola* (beach). (A) QTL plot representing LOD scores. The *X*‐axes indicate genetic distance (top) or physical distance (bottom). The two‐end arrow indicates a genomic region with suppressed recombination. (B) Structural rearrangement within the QTL region. Two‐end arrows indicate the regions corresponding to the one in (A). The arrows in the rearranged blocks indicate the corresponding order between the parents. (C) Gene‐based synteny plot in the rearranged region. Curved lines indicate orthologous pairs, while vertical bars indicate ORFs in forward (blue) and reverse (green) directions. (D) Schematic of *VmSOS1* and *VlSOS1*. Dashed lines indicate the corresponding locations between the two. (E) Expression profiles of *SOS1* and *SOS2* in the root of the four accessions. Numbers below the *X*‐axes indicate hours after initiation of salt stress. Cont (blue) and salt (orange) indicate the control and salt‐stressed conditions, respectively. *Y*‐axes indicate the abundance of transcripts in count per million (CPM). Small plots indicate values of each replicate, while larger ones and error bars indicate mean ± SD. [Color figure can be viewed at wileyonlinelibrary.com]

As *V. marina* alkalized the hydroponic culture when exposed to salt stress, we suspected that the SOS‐related genes were involved in sodium excretion. The diurnal regulation of sodium excretion as observed above further intrigued us to test whether the SOS‐related genes were also diurnally regulated or not. Thus, we performed RNA‐seq on the leaf and the root samples collected in the light period (11 am) and in the dark period (11 pm). The following clustering identified four clusters from each data set, which were diurnally upregulated or downregulated at least under the condition of salt stress (Supporting Information S1: Figure 5A).

Although the output of GO‐enrichment analysis was not highly informative (Supporting Information S1: Tables 2−9), we found that *VmSOS2* was, both in the leaf and the root, one of the diurnally‐regulated genes that were upregulated in the light period and downregulated in the dark period (Supporting Information S1: Figure 5B). In addition, those upregulated in the leaf during the dark period were enriched with GOs related to vesicle transport, which could be related to phloem transport (Supporting Information S1: Table 3).

### 3.6 Comparing Dynamics of ^22^Na Excretion Among the 4 Accessions

**Figure 6:**
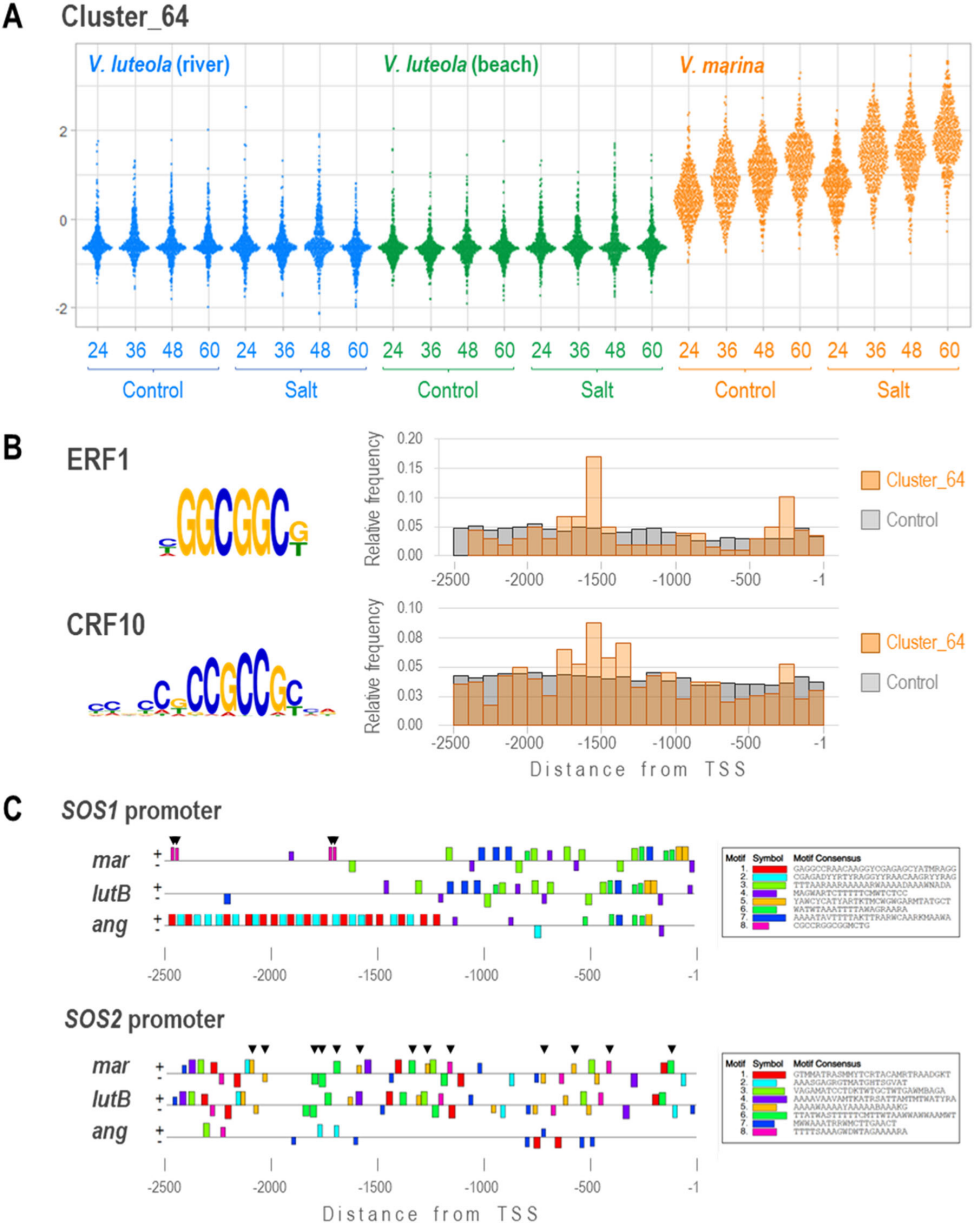
Enriched motifs in promoters (2.5 kb upstream) of *SOS1*, *SOS2* and highly expressed genes in *Vigna marina*. (A) Expression profiles of genes in cluster_64 grouped by SOM clustering. (B) Enriched motifs in the promoter sequences of the genes in cluster_64. The potentially binding transcription factors and the relative frequency of the motifs across the promoters are also shown. (C) Enriched motifs of the promoters of *SOS1* and *SOS2* loci in *V. marina*, *V. luteola* (beach) and *V. angularis*. Arrowheads indicate the motifs containing AP2/EREBP binding sites in *SOS1* promoters and those potentially involved in circadian rhythm in *SOS2* promoters. [Color figure can be viewed at wileyonlinelibrary.com]

The diurnal regulation of sodium excretion of *V. marina* led us to wonder if it was a unique feature of *V. marina*. Thus, we further performed PETIS experiments on *V. angularis*, *V. luteola* (river) and *V. luteola* (beach) along with *V. marina*. As in the former PETIS experiment, we fed the plants with ^22^Na for 24 h and then transferred to non‐^22^Na containing culture (Figure 4, Supporting Information S1: Figures 6−10 and Video).

The real‐time images by PETIS revealed a great variation across the four accessions (Figure 4, Supporting Information S1: Figures 7−10). *V. angularis* and *V. luteola* (river) retained little ^22^Na in the root, indicating most of ^22^Na had passed through the root and was transported to the shoot (see also Supporting Information S1: Figures 8 and 9). In contrast, *V. luteola* (beach) allocated the highest amount of ^22^Na in the root. *V. marina* also allocated a high amount of ^22^Na but lower than *V. luteola* (beach) did. With time, we observed in all the four accessions that the amount of ^22^Na in the root decreased while that in hydroponic culture increased (Supporting Information S1: Figures 7−10 and Video).

The following quantification of PETIS data revealed that, interestingly, the diurnal pattern of ^22^Na excretion was observed not only in *V. marina*, but in *V. luteola* (beach) and even in *V. luteola* (river) (Figure 4A). In contrast, *V. angularis* did not show any sign of the diurnal pattern (Figure 4A). At least from 12 to 36 h in the PETIS analyses, the velocity of sodium excretion during the light period was significantly larger than the dark period in *V. marina* and *V. luteola*s (Figure 4B). The excretion velocities (kBq·cm^−^^3^·h^−1^) in the light and the dark periods were 1.3 versus 0.35 in *V. marina*, 0.94 versus 0.50 in *V. luteola* (beach), 0.42 versus 0.03 in *V. luteola* (river) and 0.17 versus 0.12 in *V. angularis*, respectively. In addition, the velocities during the light period were significantly different across the accessions.

### 3.7 QTL Analysis on Salt Tolerance

To elucidate the genetic factors underlying the salt tolerance of *V. marina*, we also evaluated the degree of salt tolerance of the F_2_ plants (wilt score, shoot generation score and stay green score) (Supporting Information S1: Figure 1 and Table 10). Together with the genotype data described above (Supporting Information S1: Data), we performed a genome scan and detected two QTL peaks that were associated with all three phenotype scores, explaining ~25% of phenotypic variance (Figure 5, Supporting Information S1: Figure 11 and Table 10). In both QTLs, the alleles of *V. marina* type were associated with higher salt tolerance.

Although the LOD curves along the genetic map presented sharp peaks, those along the physical map revealed that especially the higher peak (LOD score of 9.5) ranged at least 14.5 Mbps (17.5−32.0 Mbp). Interestingly, this region was overlapped with one of the inversions between the genomes of *V. marina* and *V. luteola* (beach) (Figure 5B, Supporting Information S1: Figure 4C) and this was why recombination was highly suppressed (Figure 5A, Supporting Information S1: Data). This QTL region contained several loci potentially involved in salt tolerance, including *Salt Overly Sensitive 1* (*SOS1*), *Altered Expression of Ascorbate Peroxidase 2 8* (*ALX8*), *GASSHO1* (*GSO1*), *No Vein* (*NOV*) and *Small Rubber Particle Protein 1* (*SRP1*) (Figure 5C, Supporting Information S1: Tables 11 and 12).

The other QTL region ranged ~4 Mbp (64.3−67.6 Mbp) harbouring *SOS2, Maintenance of Photosystem II Under High Light 2* (*MPH2*), *HVS22 Homologue A* (*HVA22A*) and *Purple Acid Phosphatase 26* (*PAP26*) (Supporting Information S1: Tables 11 and 12).

As *V. marina* seemed to have higher Na^+^/H^+^ antiporter activity, we further investigated the polymorphisms of *SOS1* locus, which encodes one of the most well‐known Na^+^/H^+^ antiporters, between the parental accessions (Figure 5D). As a result, we found that four insertions of TE‐like sequences in *VmSOS1*, whereas one in *VlSOS1*. Of the five insertions, two were located in upstream of the transcription start site (TSS) of *VmSOS1*.

### 3.8 Expression Profiles of the Genes in QTLs

To identify DEGs within the QTL regions, we performed another transcriptome analysis on the four accessions. In this analysis, we started salt stress at 11:00 am on Day 0 and sampled 11:00 am (24 h) and 11:00 pm (36 h) on Day 1, and 11:00 am (48 h) and 11:00 pm (60 h) on Day 2. For *V. angularis*, we sampled only on Day 1 (24 and 36 h).

Of all the genes in *V. marina*, we first looked into the expression profiles of *SOS1* and *SOS2*, as the results described above (Figures 2, 3, 4 and 5A–D) indicated *SOS1* and *SOS2* were involved in Na^+^ excretion and its diurnal regulation. As expected, *SOS1* showed a positive correlation between the salt tolerance and the transcript abundance in the root (Figure 5E, Supporting Information S1: Table 12). In *V. angularis*, the transcript of *SOS1* was the least in the control condition and was not induced by salt stress. In *V. luteola* (river), it was ~three times higher than that of *V. angularis* in the control condition and was slightly increased in response to salt stress. In *V. luteola* (beach), it was similar to that of *V. luteola* (river) in the control condition but more strongly induced in response to salt stress. In *V. marina*, it was already high in the control condition and even increased in response to salt stress (Figure 5E). The expression pattern of *SOS1* in the leaf was similar to that in the root, except it was dramatically induced in *V. luteola* (river) in response to salt stress (Supporting Information S1: Table 11). Further, the independent qPCR analysis confirmed the expression profile of *SOS1* (Supporting Information S1: Figure 12). *SOS2* was again upregulated in the light period and downregulated in the dark period, not only in *V. marina* but also in *V. luteola* (river) and *V. luteola* (beach) (Figure 5E, Supporting Information S1: Tables 11 and 12). In contrast, there was no sign of diurnal regulation in *V. angularis* (Figure 5E).

As those in the QTL regions other than *SOS1* and *SOS2* could be responsible for the salt tolerance of *V. marina*, we performed SOM‐clustering and grouped them according to the expression patterns. As a result, *ALX8*, *NOV*, *SRP1*, *HVA22A*, *MPH2* and *PAP26* were grouped into a cluster containing *SOS1*, which were more abundantly transcribed in the root of *V. marina* than in *V. luteola*s under any conditions and timepoints (Supporting Information S1: Table 12). Of them, *SOS1, ALX8, HVA22, MPH2* and *PAP26* were also highly transcribed in the leaf of *V. marina* before salt stress, although some in *V. luteola* (river) showed a dramatic increase by salt stress (Supporting Information S1: Table 11). Though *GSO1* was not transcribed much in the control condition, it was strongly upregulated in the root by salt stress (Supporting Information S1: Tables 12).

### 3.9 Promoter Analysis on *SOS1* and *SOS2*

Because we considered the higher expression of *SOS1* is one of the key factors for the salt tolerance of *V. marina*, we investigated the promoter (2.5 kb upstream from TSS) of *SOS1* locus for enriched motifs.

First, as the *VmSOS1* had a TE insertion at the site of −1267 bp from TSS, we screened the inserted sequence for enriched motifs. The results revealed ‘CGCCRGGCGGMCTG’ as enriched, which could potentially be a binding site for AP2/EREBP transcription factors such as ERF1 and CRF10 (Figures 5D and 6B,C).

To test if this motif is associated with the expression profile of *VmSOS1*, we extracted the promoter sequences from those genes highly expressed in *V. marina* but not in *V. luteola*s (cluster_64) and from other genes as control (Figure 6A). Compared to the control genes, the promoters of genes in cluster_64 were significantly more enriched with the potential binding sites for ERF1 (0.48/2.5 vs. 0.30/2.5 kb) and CRF10 (0.94/2.5 vs. 0.82/2.5 kb). In addition, the ERF1 motifs were highly enriched around 0.2−0.3 and 1.5−1.7 kb upstream of those in cluster_64, whereas it showed a more flat distribution in the promoters of the control genes (Figure 6B). The CRF10 motifs were also enriched in 1.3−1.7 kb upstream of the cluster_64 genes (Figure 6B).

Finally, we compared the promoter sequences of *SOS1* and *SOS2* across *V. marina*, *V. luteola* (beach) and *V. angularis*. The motif scan revealed that the *V. angularis* conserved very few motifs, except the first ~400 bp upstream of the *SOS1* TSS (Figure 6C), whereas *V. marina* and *V. luteola* (beach) highly conserved the promoter structures except the TE insertion (Figures 5D and 6C). Of the four ‘CGCCRGGCGGMCTG’ motifs in the *VmSOS1* promoter, two were placed at 1689 and 1711 bp upstream of TSS, as in many of the cluster_64 genes (Figures 5D and 6B,C). The result also revealed that the promoters of *VmSOS2* and *VlSOS2* harboured 13 potential binding sites for *B‐box domain protein 31* (*BBX31*), which is involved in ‘circadian rhythm’, whereas *VaSOS2* harboured no motifs of such kind (Figure 6C).

## 4 Discussion

Here, we revealed not only that *V. marina* has a greater ability to excrete sodium out of the root but also that sodium excretion is under diurnal regulation. As sodium excretion must occur against the direction of water uptake, it was surprising to observe that *V. marina* excreted sodium mainly during the light period, which is usually the time of transpiration. In addition, the forward genetic and transcriptome analyses indicate that *SOS1* could determine the maximum rate of sodium excretion, and *SOS2* could regulate its diurnal manner. The upregulation of *SOS1* could be due to the TE insertion in the promoter, which might have installed binding motifs for AP2/EREBP transcription factors. The diurnal regulation of *SOS2* could also be explained with its promoter sequences, which had been acquired before the divergence of *V. marina* and *V. luteola*.

### 4.1 Diurnal Regulation of Sodium Excretion

It was surprising to discover by PETIS that *V. marina* and *V. luteola* excrete sodium in a diurnally‐regulated manner: more in the light period and less in the dark period (Figures 3 and 4). The diurnal pattern of ^22^Na in the shoot was not surprising, as sodium is usually transported to the shoot in a transpiration‐dependent manner (Dinneny 2015) and recirculated to the root via phloem transport mediated by vesicle transport (Berthomieu and Casse 2003). The recirculation is supported by our results that the vesicle transport genes were upregulated in the leaf during the dark period (Supporting Information S1: Table 3). The decrease of root sodium during the light period could also be, to some extent, due to transpiration‐dependent transport to the shoot. However, as the sodium concentration in hydroponic culture also increased in the light period, the decrease in the root was also due to sodium excretion. If the outward transport were a passive diffusion, it should be slow in the light period and fast in the dark period, given the transport must occur against the direction of water uptake. As the result was the opposite (Figures 3 and 4), *V. marina* is able to actively excrete sodium from the root during the light period.

It was first puzzling to notice that the diurnal regulation of SOS‐dependent sodium excretion is conserved across species, given high and low tides do not occur in 24 h cycle, or extreme high tides by typhoons could occur at any time of a day. However, if the SOS pathway is energetically costly, the diurnal downregulation could contribute to minimizing the negative effect on growth. As the transpiration‐dependent sodium uptake does not occur during the dark period, it could be reasonable to activate the SOS pathway only in the light period (Figure 3).

### 4.2 Role *SOS1* and *SOS2* in Sodium Excretion of *V. marina*

As the sodium excretion was accompanied by alkalization of hydroponic culture (Figure 2), we consider the Na^+^/H^+^ antiporter activity of *SOS1* (Ismail and Horie 2017) plays the key role in excreting sodium out of the root. This hypothesis is also supported by our results that *SOS1* is located in the highest QTL of salt tolerance (Figure 5) and that *SOS1* transcription was highly correlated not only with salt tolerance but the ability of sodium excretion (Figures 3, 4, 5). In addition, the pattern of *SOS1* expression also well explains the manner of salt allocation observed in this study (Figure 1A) and our previous study (Noda et al. 2022): The lower expression of *SOS1* in the root of *V. angularis* and *V. luteola* (river) cannot suppress sodium loading to xylem and allow high allocation to the shoot. In contrast, the higher expression of *SOS1* in the root of *V. marina*, even before salt stress, enables immediate sodium excretion after initiation of salt stress. Although the expression of *SOS1* in the root of *V. luteola* (beach) is strongly induced, the lower expression in the control condition might allow sodium to accumulate in the root in the early stage of salt stress.

The diurnally‐regulated transcription of *SOS2*, encoding calcineurin binding protein‐like (CBL) interacting protein kinase (CIPK) that directly phosphorylates and activates SOS1 (Liu et al. 2000), also explains the observed pattern of sodium excretion. Though we have found only a correlation, but *V. marina* and *V. luteola*s exhibited a diurnal pattern in *SOS2* transcription and sodium excretion, while *V. angularis* did neither in SOS2 transcription nor sodium excretion (Figures 3 and 5). However, although *VmSOS2* was also located within one of the QTLs, it could not explain the difference of salt tolerance between *V. marina* and *V. luteola* (beach) given its transcriptional pattern was not different (Figure 5).

### 4.3 Evolution of Transcriptional Regulation in *SOS1* and *SOS2*

Our results suggested that the upregulation of *VmSOS1* is a new example of gene regulatory network rewiring by TE insertions (Feschotte 2008; Chuong, Elde, and Feschotte 2017). The TE insertion at −1207 bp position of *VmSOS1* placed the AP2/EREBP motif at −1689 bp (Figures 5D and 6C), and such motifs were also enriched around −1.5 kbp positions of the genes highly expressed in *V. marina* (Figure 6). Though what we found is currently only a correlation, it is an intriguing hypothesis to test whether the TE has installed the key *cis*‐regulatory element to the *SOS1* promoter.

The output of our promoter analysis also suggested that *V. marina* and *V. luteola* have acquired a set of *cis*‐regulatory elements for diurnal regulation before they have diverged from the common ancestor. The structure of *VaSOS2* promoter, which was totally different from *VmSOS2* and *VlSOS2*, could also explain why *VaSOS2* is not regulated in the diurnal manner (Figure 5E). As the diurnal expression of *SOS2* is also conserved in *V. luteola* (river), it should have played important roles in adaptation, not only to high salinity but also to other environmental cues.

Although the SOS pathway in model plants is also diurnally regulated (Park et al. 2016; Soni et al. 2013; Kim et al. 2013), the results in this study are different from those in the preceding studies. First, the transcription of *SOS1* does not seem to be diurnally regulated in Vigna species (Figure 5E). In Arabidopsis, *SOS1* transcription is more in the light period and less in the dark period (Park et al. 2016), whereas in rice it is more transcribed in the dark period (Soni et al. 2013). Second, at least in *V. marina*, the activity of the SOS pathway is maximized in the early morning (Figures 3 and 4), whereas it should be higher in the dark period in Arabidopsis and rice. In rice, *SOS2* is, together with *SOS1*, more transcribed in the dark period (Soni et al. 2013). In Arabidopsis, although there are few literatures reporting transcriptional regulation of *SOS2*, SOS2 protein is known to physically interact with GIGANTEA (GI), which is expressed during daytime, and to be prevented from activating SOS1 (Kim et al. 2013). Although GI is degraded by salt stress, it is a time‐consuming process and thus negatively affects the SOS1 activity. Therefore, the daytime‐oriented activation of the SOS pathway could be a unique feature of *V. marina* and *V. luteola*.

### 4.4 Role of Chromosome Rearrangement in Fixing the Adaptive Haplotype Within the QTL

Although we consider *SOS1* is the most important gene for the *V. marina*'s ability to excrete sodium, there are several other genes that could be involved in salt tolerance and highly expressed or salt‐induced in *V. marina* (Figure 5C, Supporting Information S1: Tables 12). As *GSO1* (Pfister et al. 2014) and *NOV* (Tsugeki et al. 2009) are involved in the development of Casparian strip and endodermis, respectively, they might contribute to developing the thick apoplastic barrier in the root of *V. marina* that effectively suppresses sodium loading to xylem (Wang et al. 2025). *SRP1* has dual roles in enhancing not only tissue growth but tolerance to abiotic stresses (Kim et al. 2016). Thus, within the QTL regions, *V. marina* might have a set of adaptive alleles across multiple gene loci.

What is interesting in our findings is that the QTL region contains structural rearrangement spanning more than 14 Mbp (Figure 5B,C, Supporting Information S1: Figure 4). As this rearrangement almost completely suppressed recombination across the whole QTL region, more than 300 gene loci behaved like a single genetic locus in our mapping population (Figure 5A−C, Supporting Information S1: Data). Such a large rearrangement could contribute to chromosome speciation, as rearranged chromosomes make the favourable allele combinations resilient to outcross (reviewed by Faria and Navarro 2010). Without the rearrangement, the linkage of adaptive alleles would be easily broken and the progenies are likely to inherit less favourable set of alleles compared to the parental genotypes.

### 4.5 Limitations

Though we have demonstrated the importance of *SOS1* and *SOS2* in *V. marina* and *V. luteola*, we have not thoroughly investigated the second QTL for the genes that could explain the parental difference in salt tolerance. The possible candidates are *HVA22A*, *PAP26*, *MPH2* and so on. *HVA22* is often strongly induced by stress and ABA, but its functional role in stress tolerance is not yet clear. *PAP26* is a member of purple acid phosphatase and enhances salt tolerance when overexpressed (Abbasi‐Vineh, Sabet, and Karimzadeh 2021). It does so by enhancing ion homoeostasis via the SOS pathway, ROS scavenging ability and acid phosphatase activity required for increasing available P storage. *MPH2* is required for repairing photosystem II (PSII) under fluctuating light conditions or photoinhibitory light (Liu and Last 2017). As PSII is particularly vulnerable to abiotic stresses including salt stress (Jajoo 2013), the higher expression of *MPH2* (Supporting Information S1: Table 12) could help acclimation of *V. marina* to saline environments. However, further studies are certainly needed to test whether these genes or others, are responsible for *V. marina*'s salt tolerance.

## 5 Conclusion

We revealed at least one aspect of the mechanism of salt tolerance in *V. marina*, which lives in marine beach and is the most salt‐tolerant species in the genus. It has a higher potential of sodium excretion from the root, presumably through the elevated expression of *VmSOS1*. In addition, sodium excretion is diurnally regulated via diurnal transcriptional regulation of *VmSOS2*, which could save the energy cost of the SOS pathway. This knowledge will be valuable information for future crop development, especially for cropping with saline water and soil.

## Conflicts of Interest

The authors declare no conflicts of interest.

## Supporting information

Supporting information.

Supporting information.

Supporting information.

Supporting information.

## Data Availability

The data that support the findings of this study are openly available in Genomic and transcriptomic data of *V. marina* and *V. luteola* at https://www.ncbi.nlm.nih.gov/bioproject/1080052, reference number PRJNA1080052. Genome assemlies are also available at https://viggs.dna.affrc.go.jp/download.
