# Supplementary material for: Diurnal Regulation of SOS Pathway and Sodium Excretion Underlying Salinity Tolerance of Vigna marina
Source: Plant Cell Environ. 2025 Jan 24;48(6):3925–38. doi: 10.1111/pce.15402 (PMC12050389; doi:10.1111/pce.15402)
Supplement: Supplementary file 1 — Supporting information. [file PCE-48-3925-s004.pptx]

## Slide 1
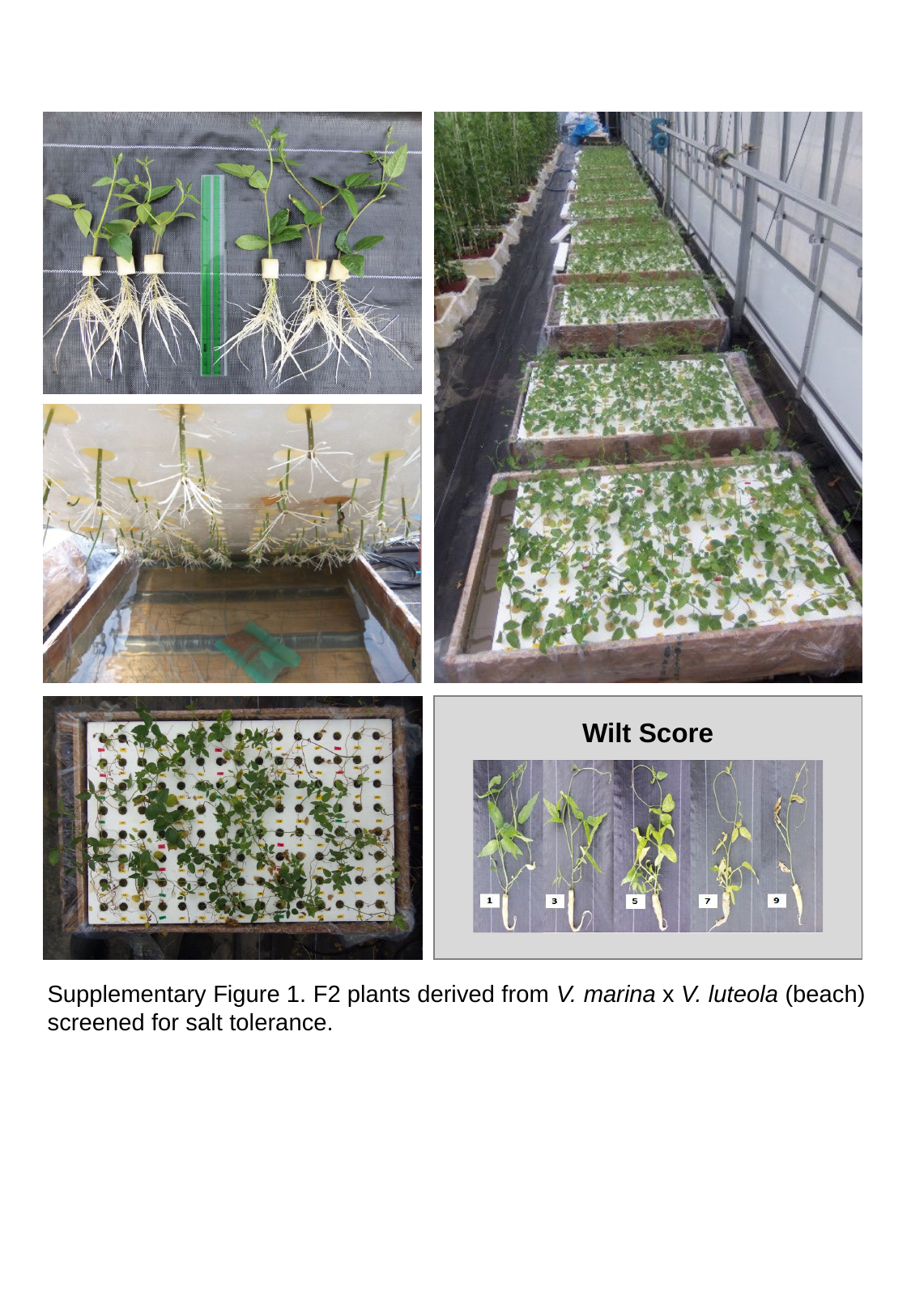

Wilt Score
Supplementary Figure 1. F2 plants derived from V. marina x V. luteola (beach) screened for salt tolerance.

## Slide 2
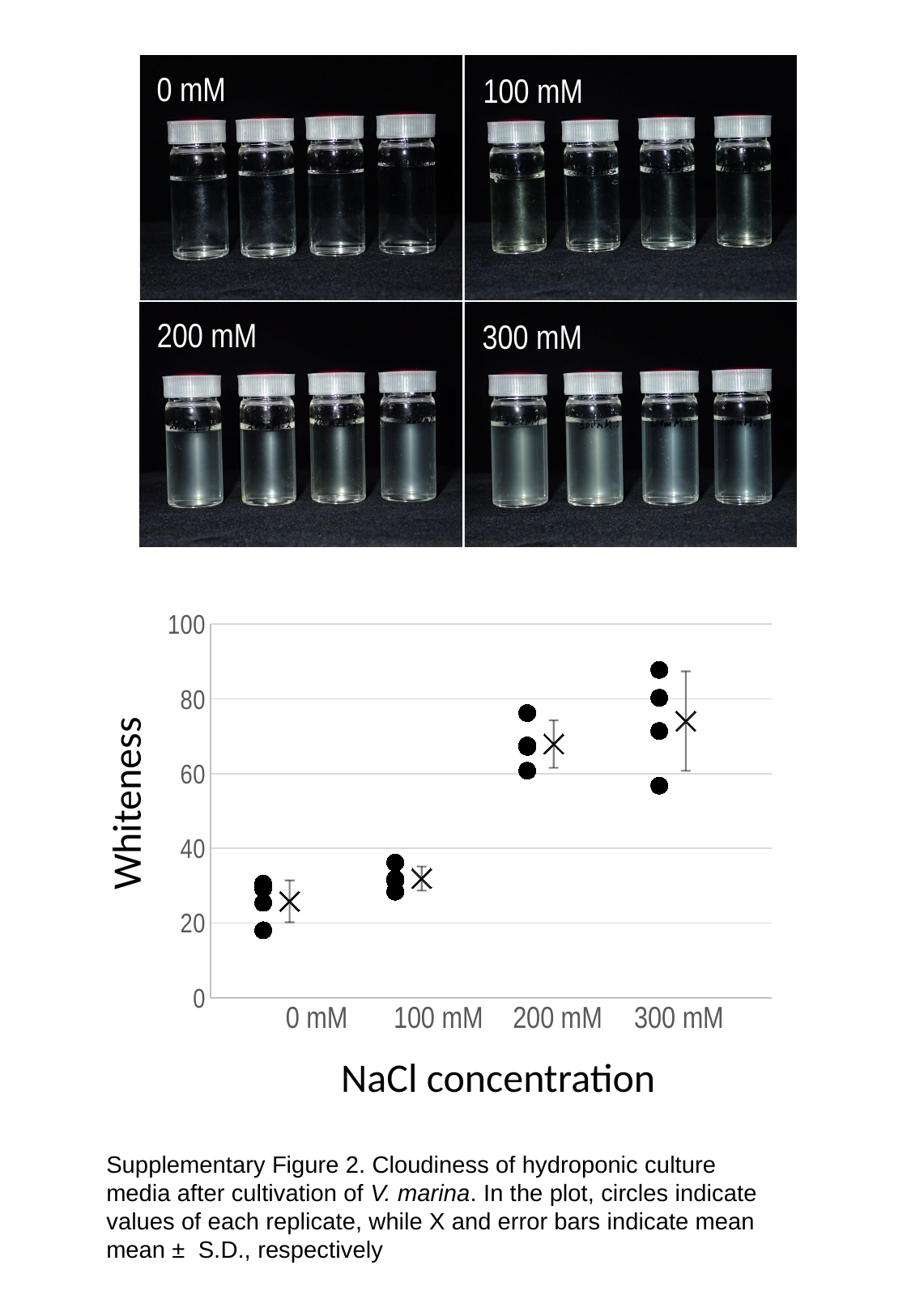

0 mM
100 mM
200 mM
300 mM
### Chart
| Category | | |
|---|---|---|Whiteness
0 mM
100 mM
200 mM
300 mM
NaCl concentration
Supplementary Figure 2. Cloudiness of hydroponic culture media after cultivation of V. marina. In the plot, circles indicate values of each replicate, while X and error bars indicate mean mean ± S.D., respectively

## Slide 3
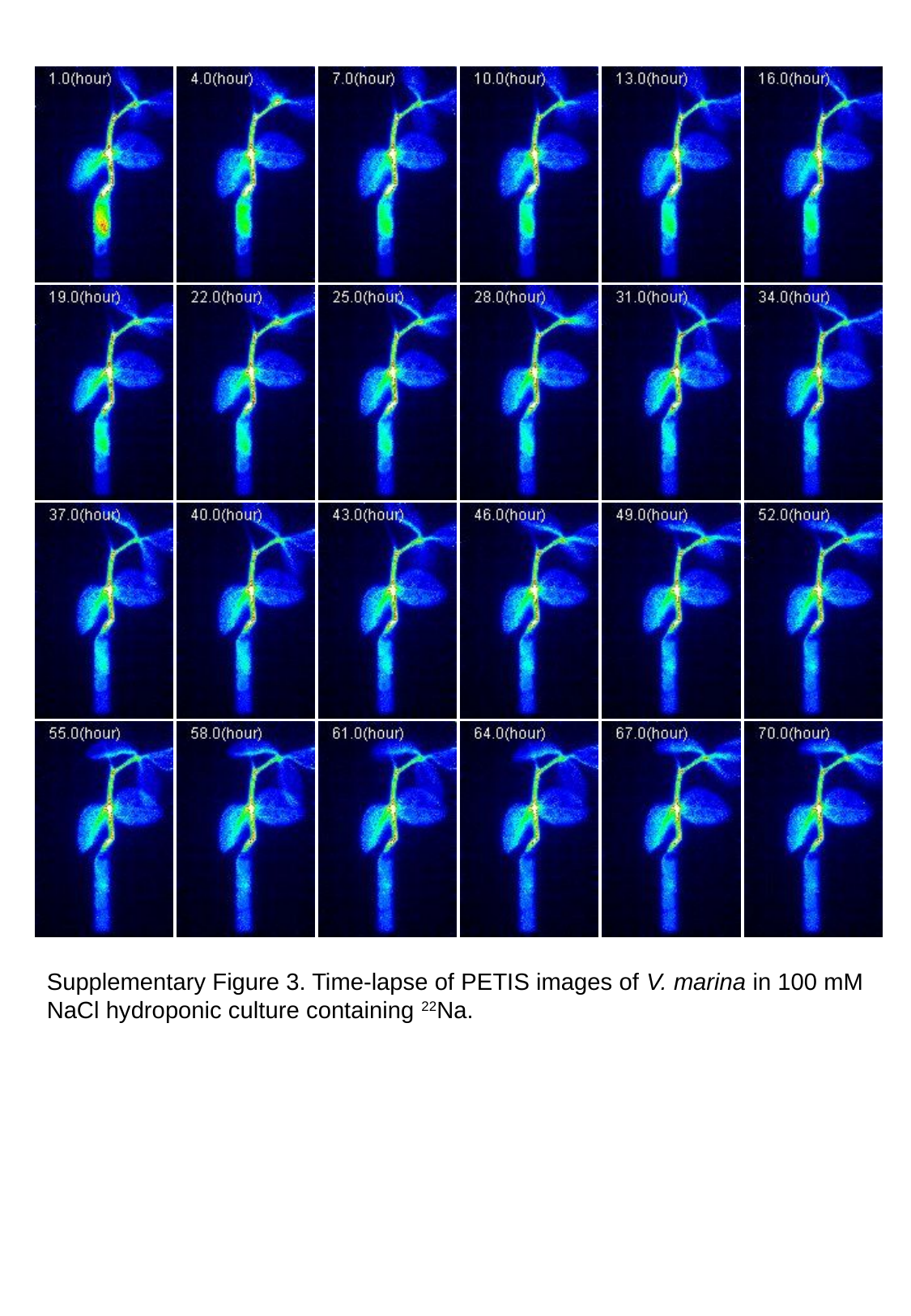

Supplementary Figure 3. Time-lapse of PETIS images of V. marina in 100 mM NaCl hydroponic culture containing 22Na.

## Slide 4
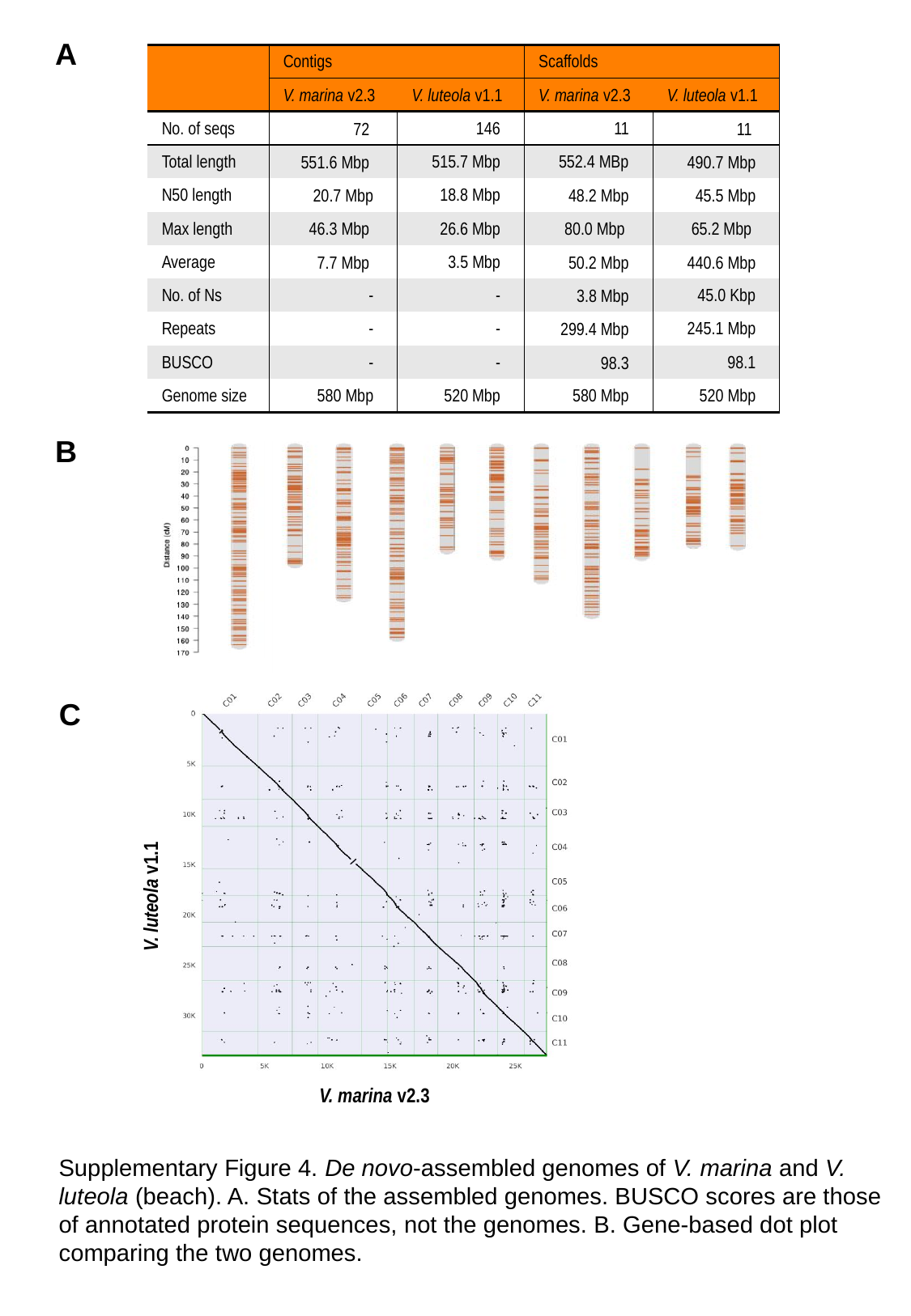

A
| | Contigs | | Scaffolds | |
| --- | --- | --- | --- | --- |
| | V. marina v2.3 | V. luteola v1.1 | V. marina v2.3 | V. luteola v1.1 |
| No. of seqs | 72 | 146 | 11 | 11 |
| Total length | 551.6 Mbp | 515.7 Mbp | 552.4 MBp | 490.7 Mbp |
| N50 length | 20.7 Mbp | 18.8 Mbp | 48.2 Mbp | 45.5 Mbp |
| Max length | 46.3 Mbp | 26.6 Mbp | 80.0 Mbp | 65.2 Mbp |
| Average | 7.7 Mbp | 3.5 Mbp | 50.2 Mbp | 440.6 Mbp |
| No. of Ns | - | - | 3.8 Mbp | 45.0 Kbp |
| Repeats | - | - | 299.4 Mbp | 245.1 Mbp |
| BUSCO | - | - | 98.3 | 98.1 |
| Genome size | 580 Mbp | 520 Mbp | 580 Mbp | 520 Mbp |
B
C
V. luteola v1.1
V. marina v2.3
Supplementary Figure 4. De novo-assembled genomes of V. marina and V. luteola (beach). A. Stats of the assembled genomes. BUSCO scores are those of annotated protein sequences, not the genomes. B. Gene-based dot plot comparing the two genomes.

## Slide 5
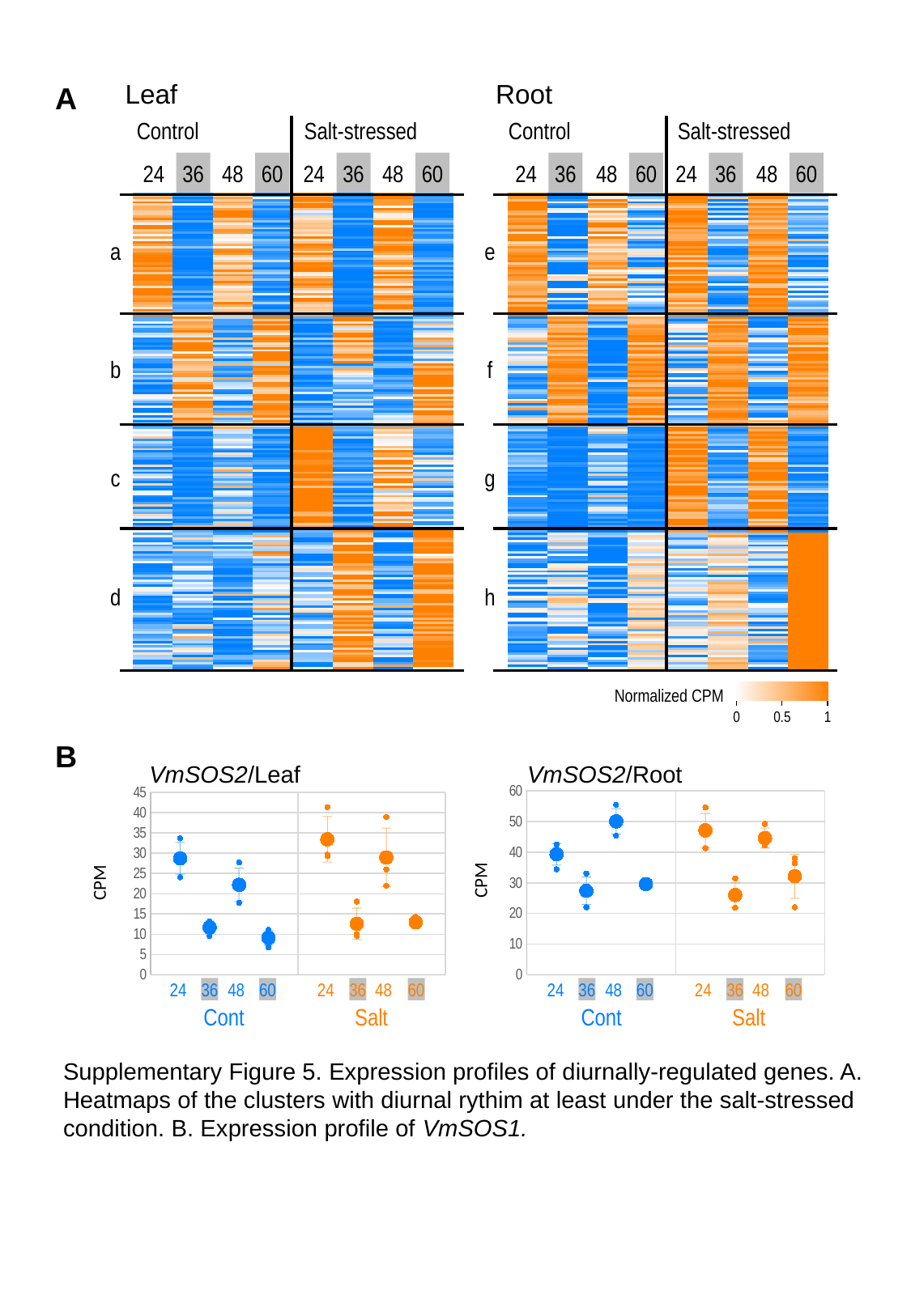

Leaf
Root
A
Control
Salt-stressed
Control
Salt-stressed
24
36
48
60
24
36
48
60
24
36
48
60
24
36
48
60
a
e
b
f
c
g
d
h
Normalized CPM
0
0.5
1
B
VmSOS2/Leaf
VmSOS2/Root
### Chart
| Category | | | | |
|---|---|---|---|---|
### Chart
| Category | | | | |
|---|---|---|---|---|CPM
CPM
24
36
48
60
24
36
48
60
Cont
Salt
24
36
48
60
24
36
48
60
Cont
Salt
Supplementary Figure 5. Expression profiles of diurnally-regulated genes. A. Heatmaps of the clusters with diurnal rythim at least under the salt-stressed condition. B. Expression profile of VmSOS1.

## Slide 6
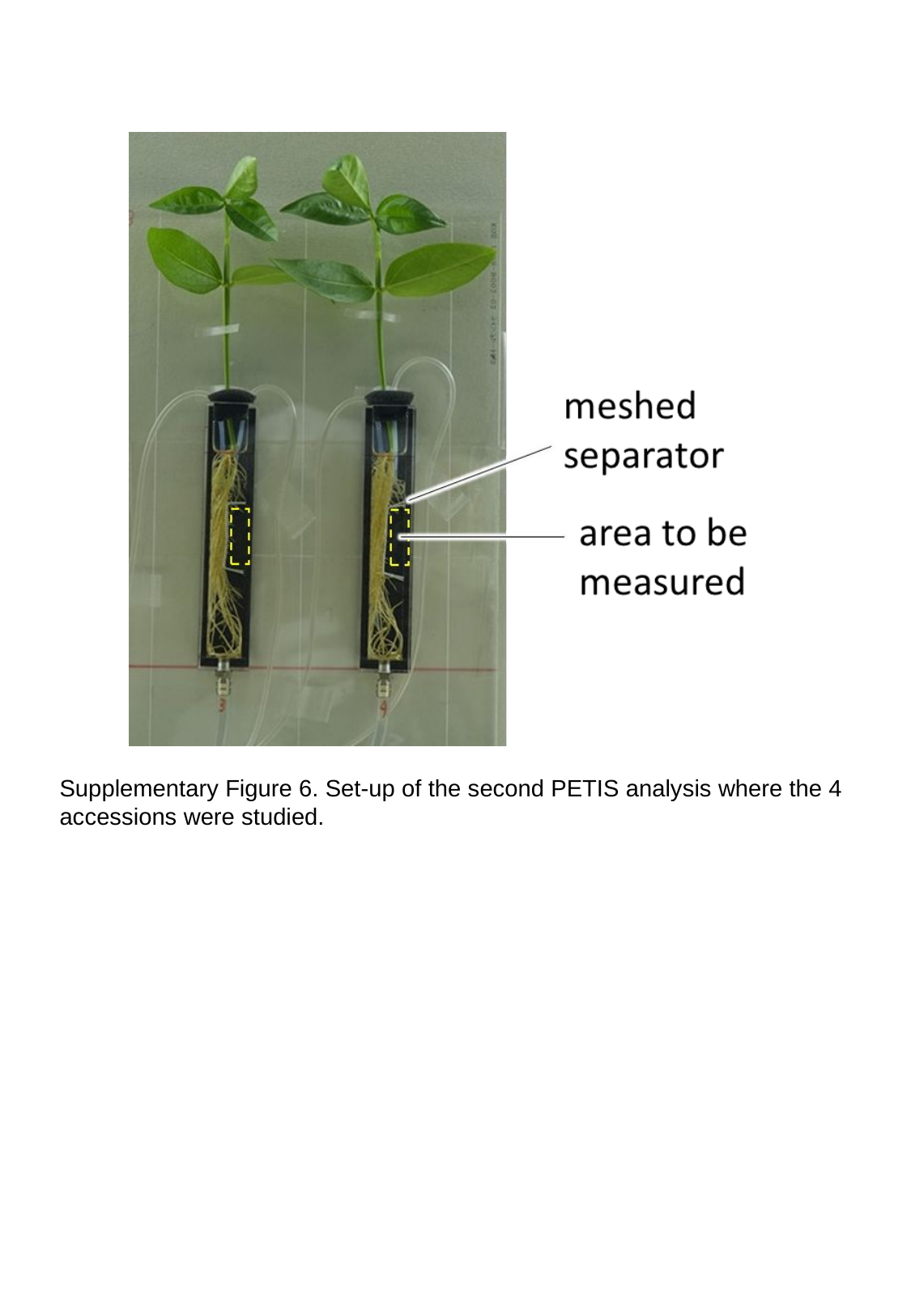

Supplementary Figure 6. Set-up of the second PETIS analysis where the 4 accessions were studied.

## Slide 7
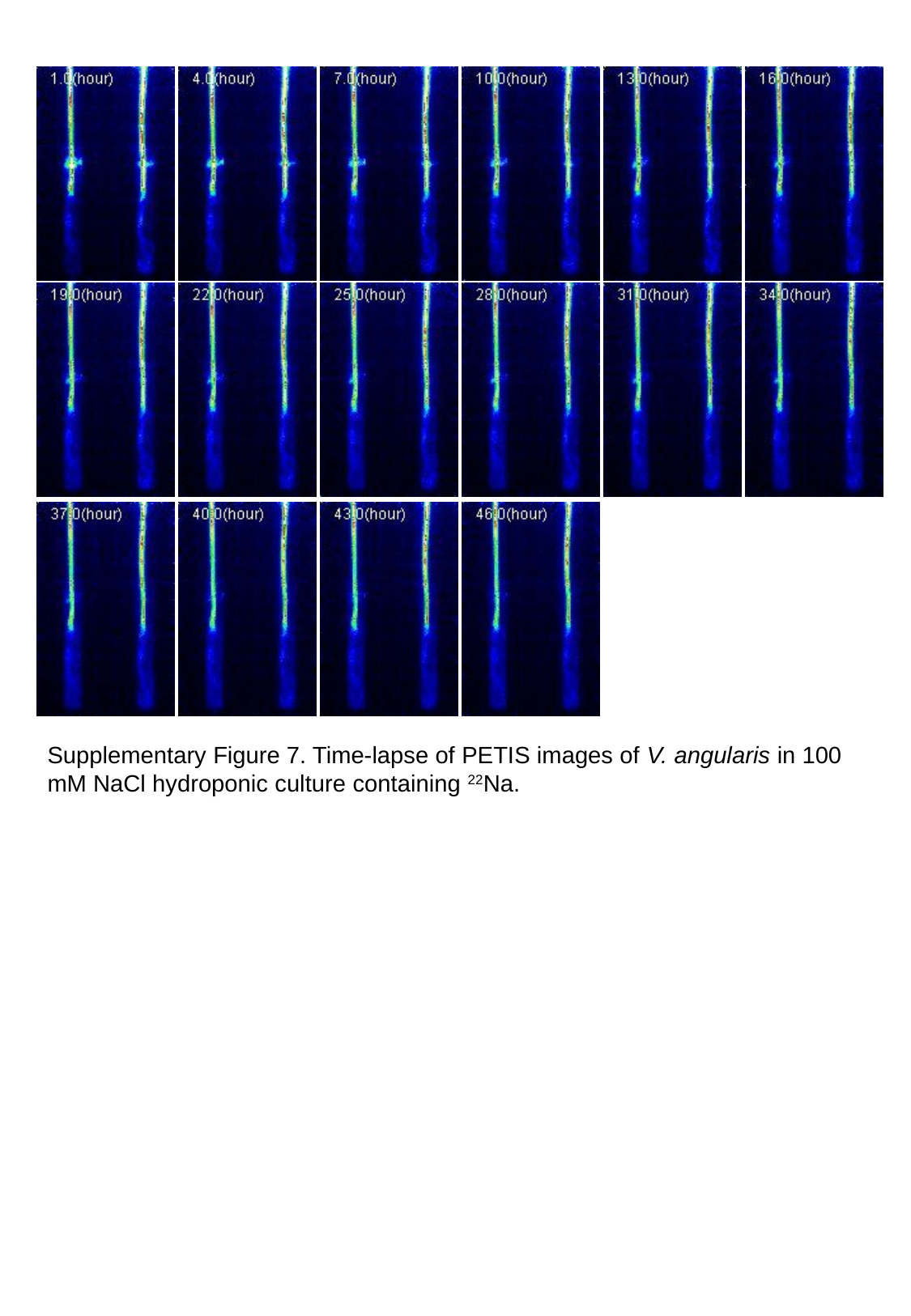

Supplementary Figure 7. Time-lapse of PETIS images of V. angularis in 100 mM NaCl hydroponic culture containing 22Na.

## Slide 8
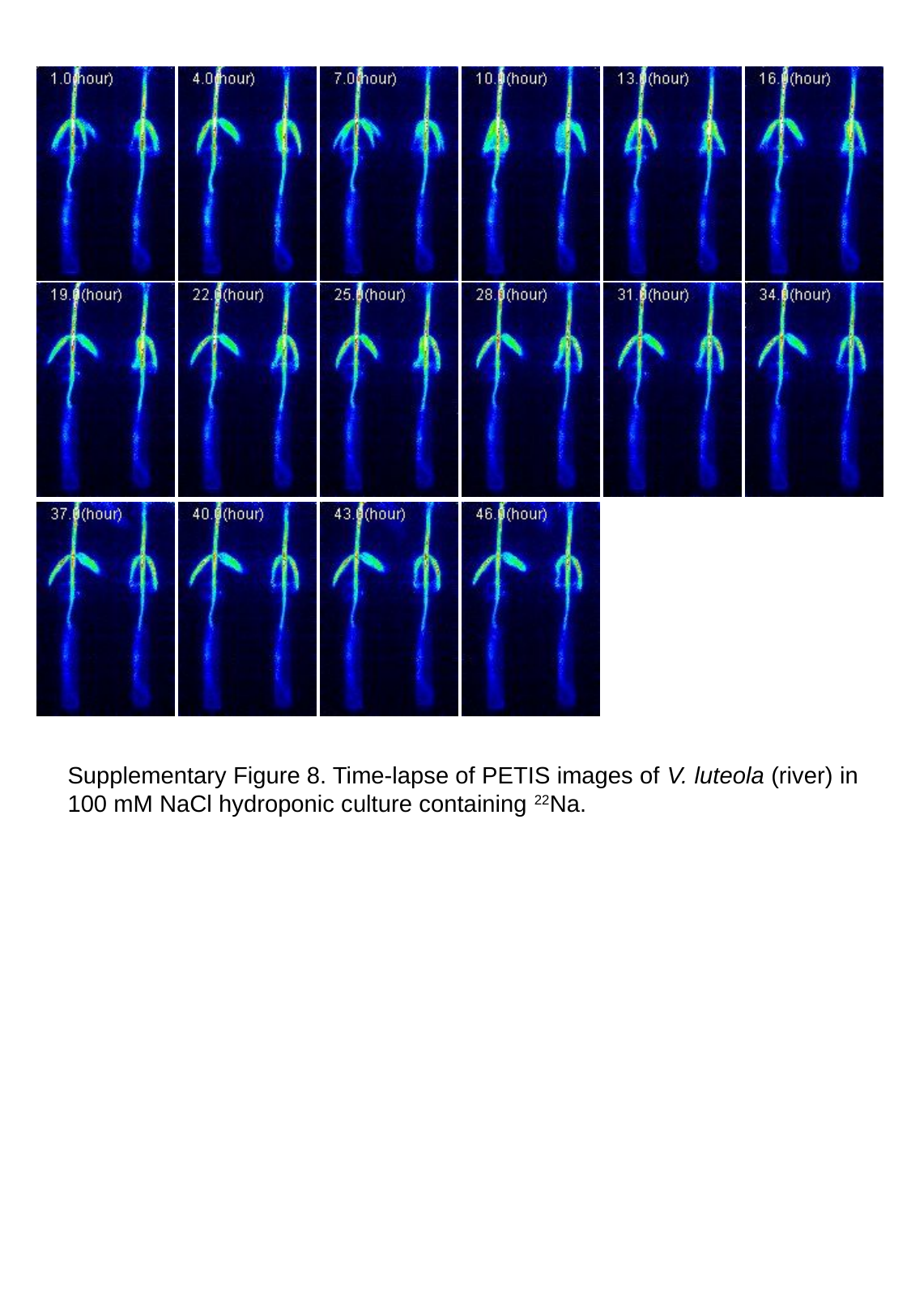

Supplementary Figure 8. Time-lapse of PETIS images of V. luteola (river) in 100 mM NaCl hydroponic culture containing 22Na.

## Slide 9
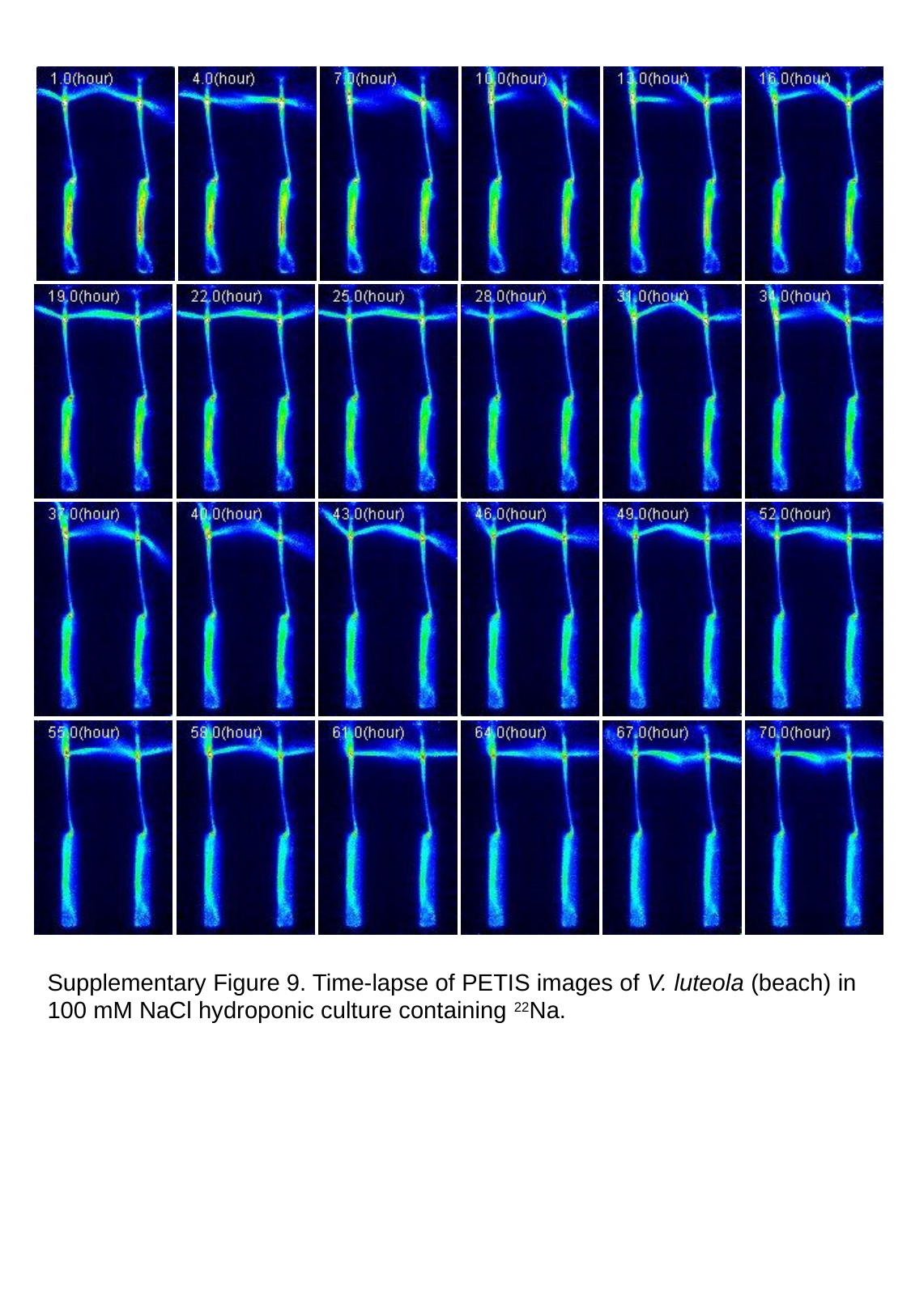

Supplementary Figure 9. Time-lapse of PETIS images of V. luteola (beach) in 100 mM NaCl hydroponic culture containing 22Na.

## Slide 10
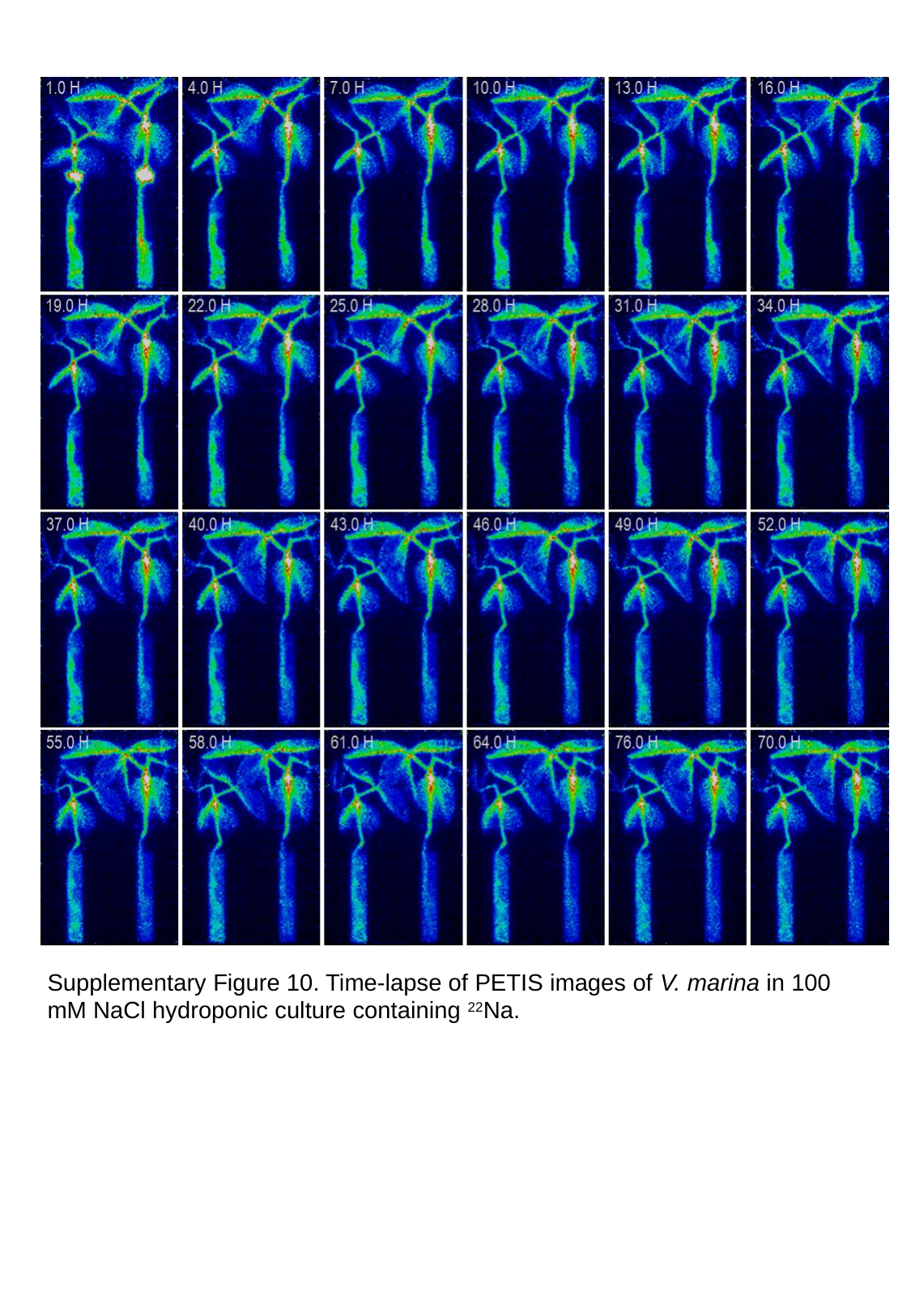

Supplementary Figure 10. Time-lapse of PETIS images of V. marina in 100 mM NaCl hydroponic culture containing 22Na.

## Slide 11
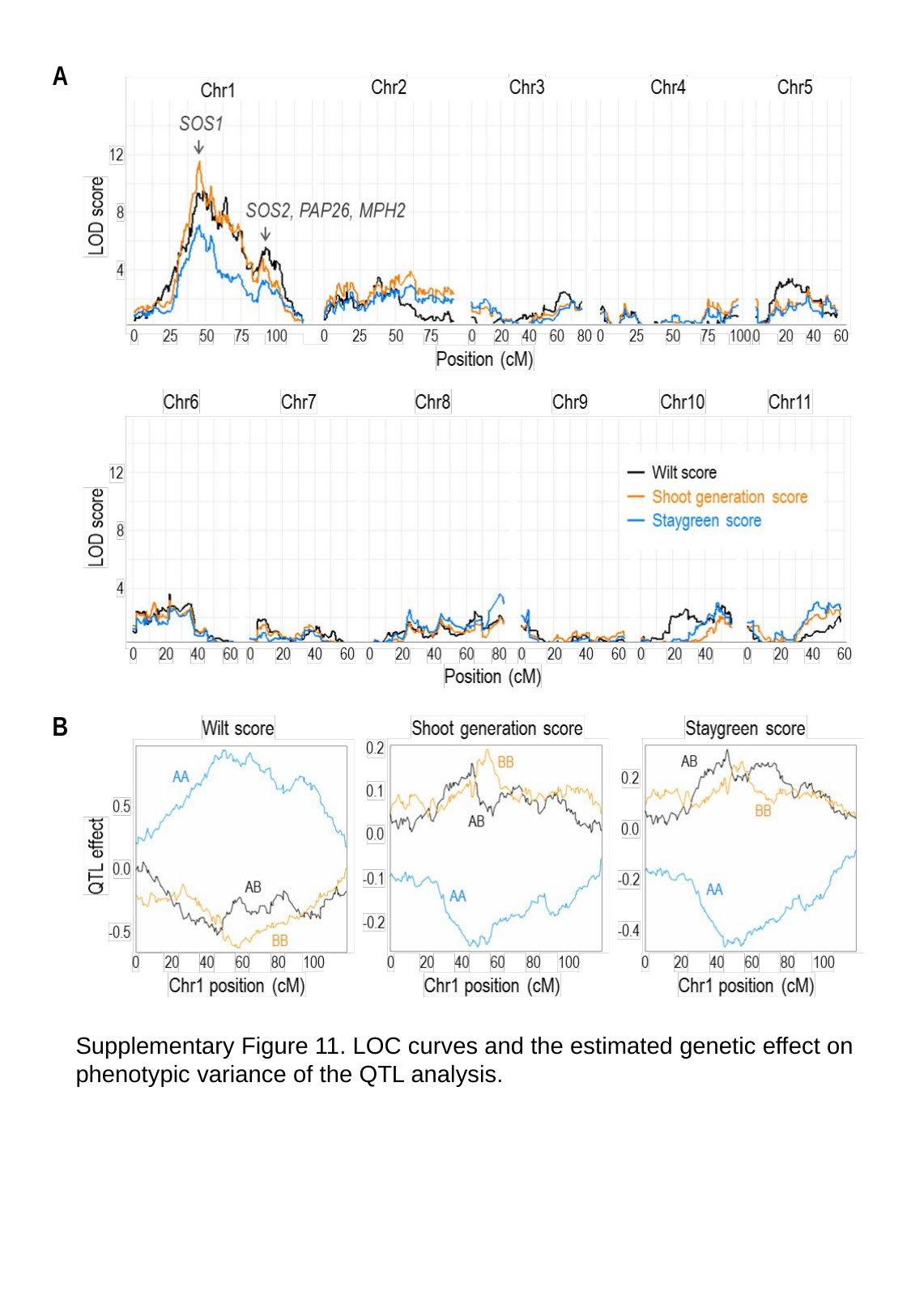

A
B
Supplementary Figure 11. LOC curves and the estimated genetic effect on phenotypic variance of the QTL analysis.

## Slide 12
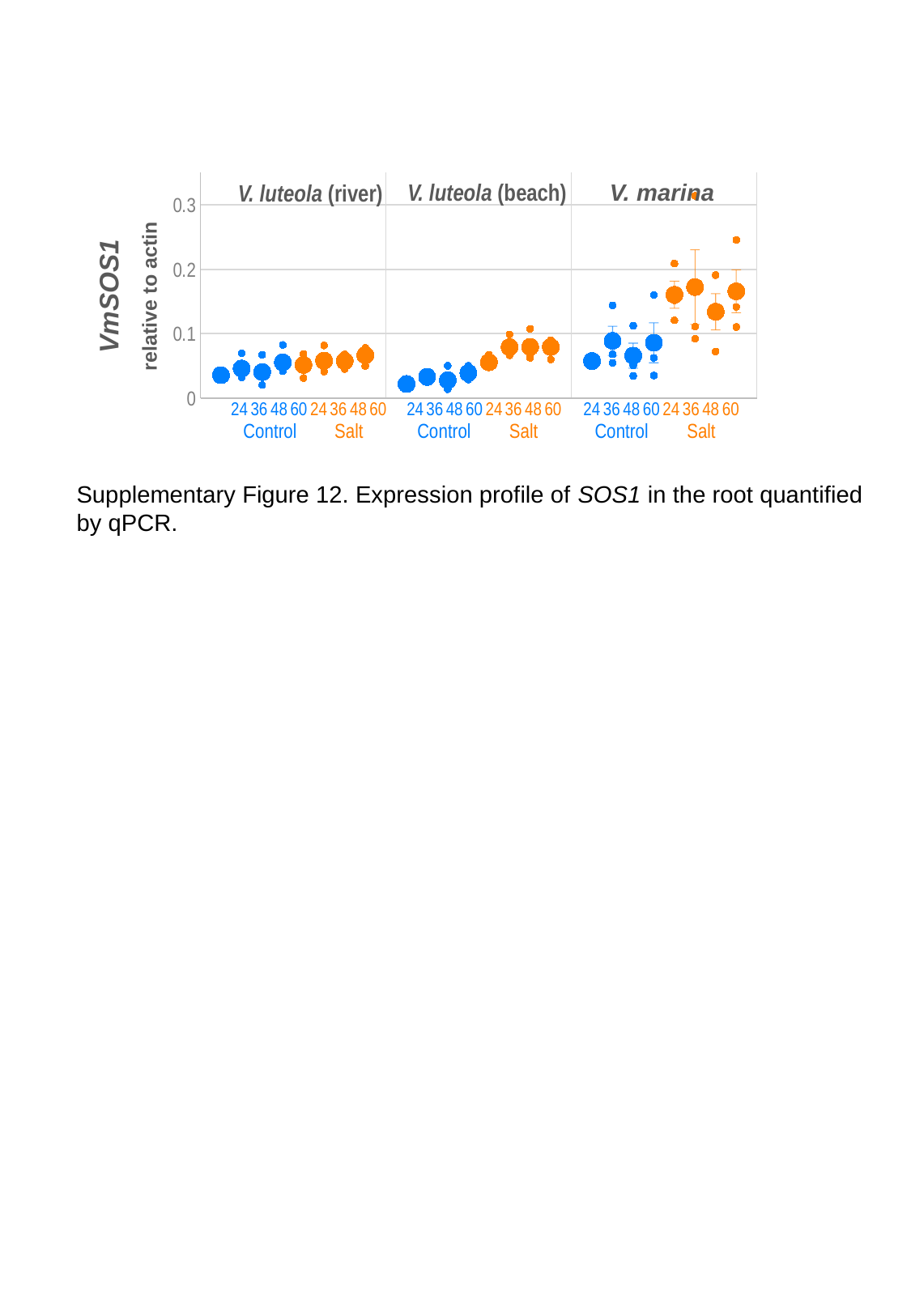

### Chart
| Category | | | | |
|---|---|---|---|---|V. marina
V. luteola (beach)
V. luteola (river)
VmSOS1
relative to actin
24
36
48
60
24
36
48
60
24
36
48
60
24
36
48
60
24
36
48
60
24
36
48
60
Control
Salt
Control
Salt
Control
Salt
Supplementary Figure 12. Expression profile of SOS1 in the root quantified by qPCR.
