# Supplementary figures and images for: Diurnal Regulation of SOS Pathway and Sodium Excretion Underlying Salinity Tolerance of Vigna marina
Source: Plant Cell Environ. 2025 Jan 24;48(6):3925–38. doi: 10.1111/pce.15402 (PMC12050389; doi:10.1111/pce.15402)

## Slide 1
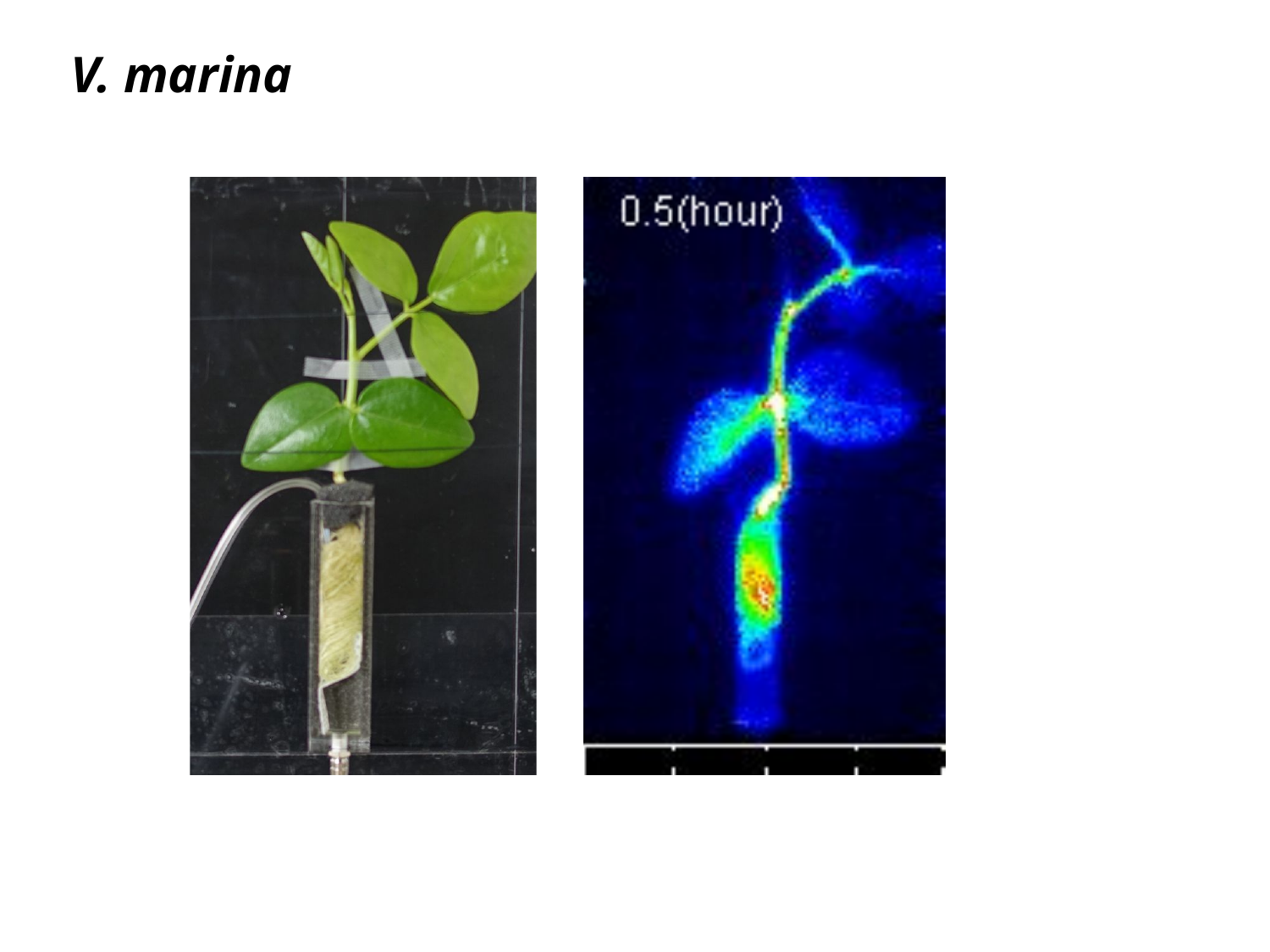

V. marina

## Slide 2
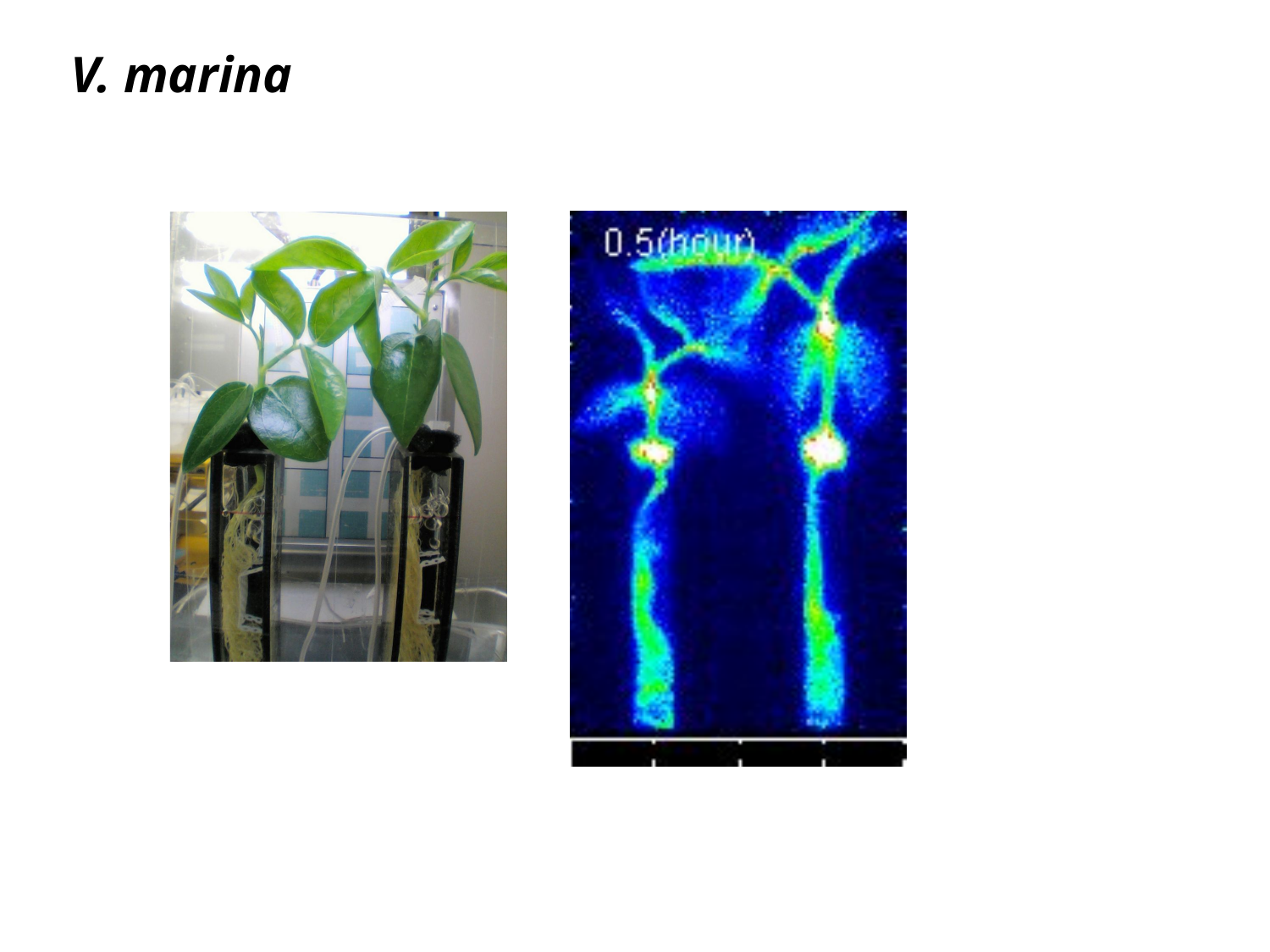

V. marina

## Slide 3
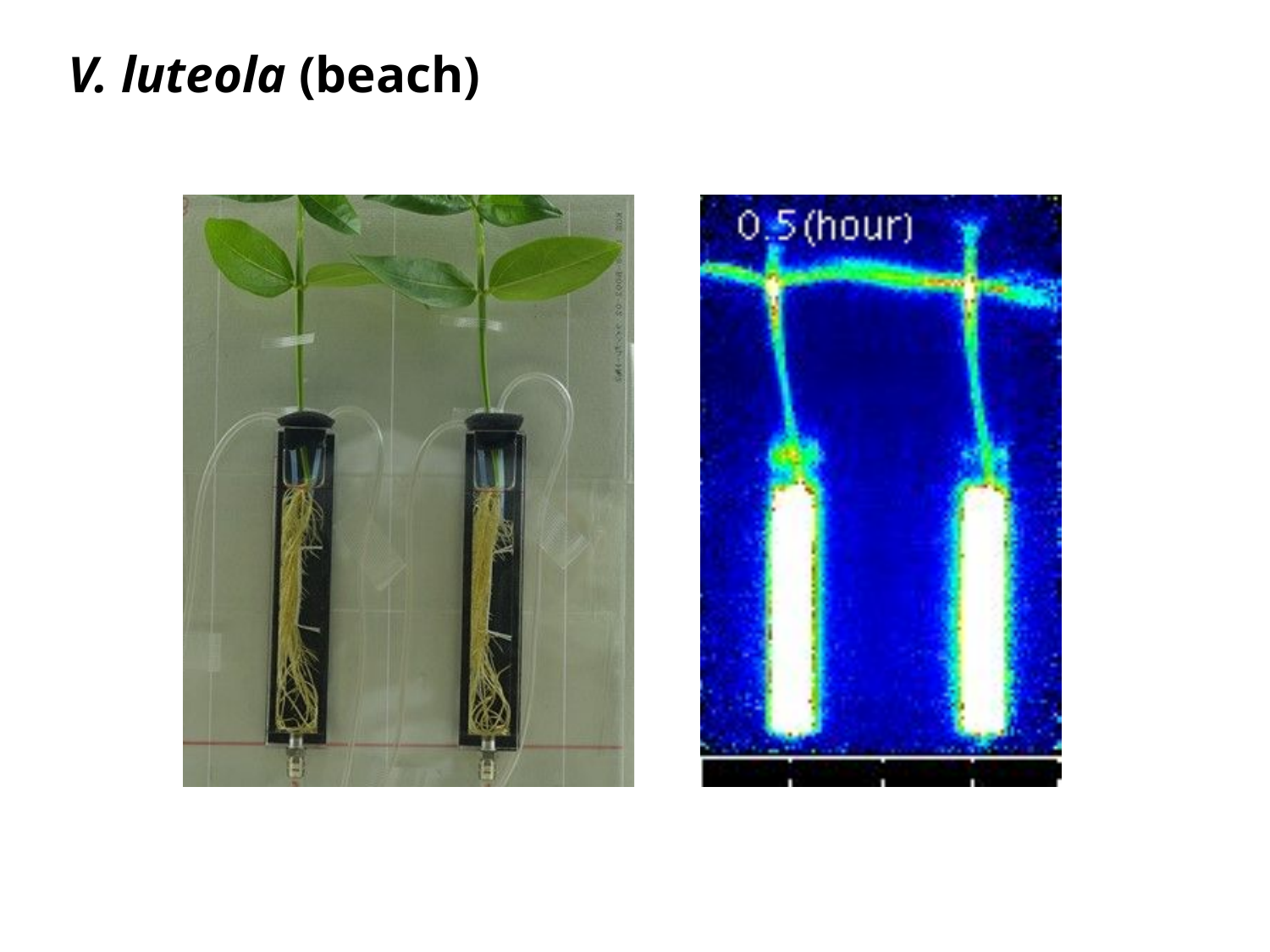

V. luteola (beach)

## Slide 4
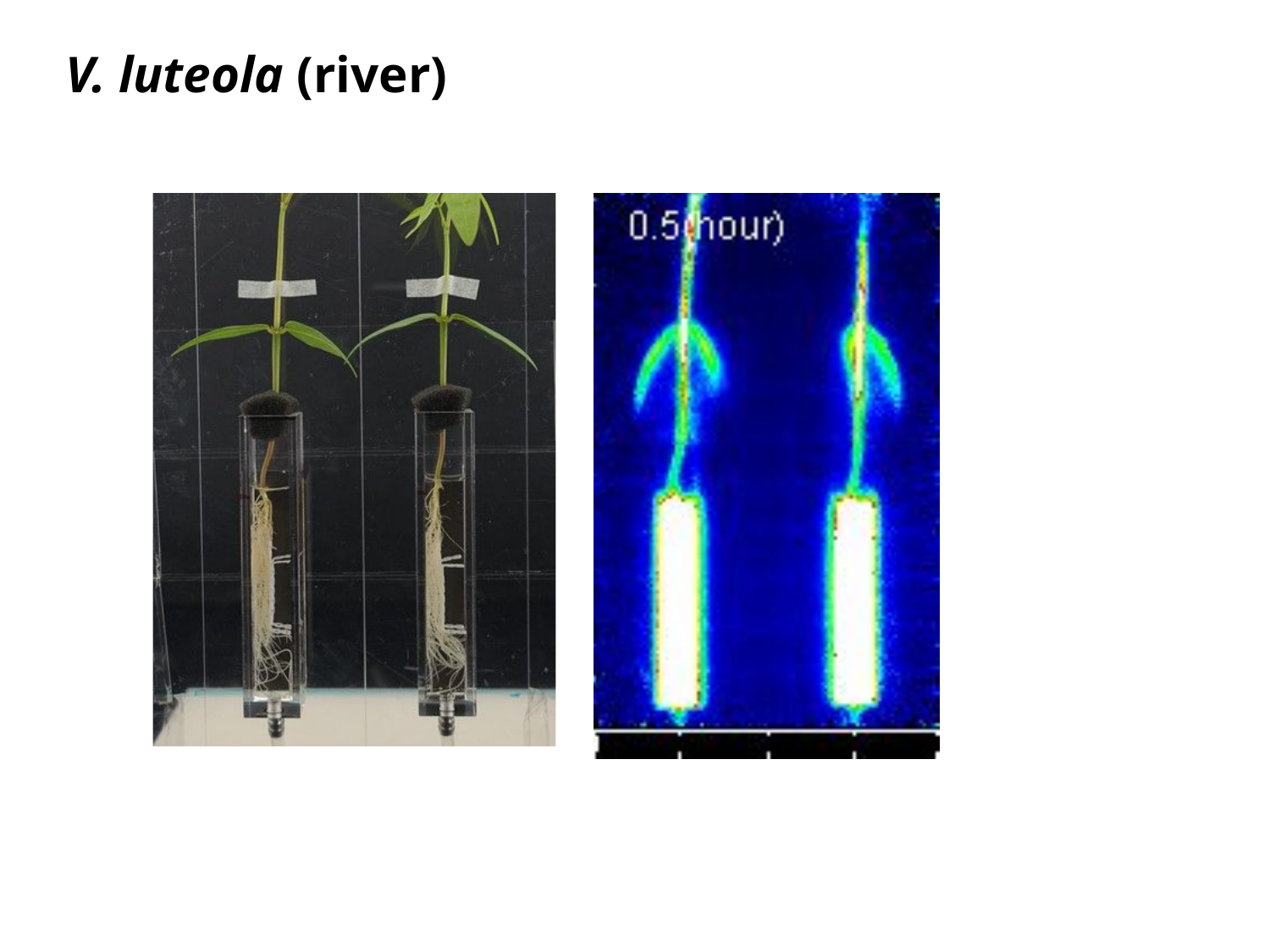

V. luteola (river)

## Slide 5
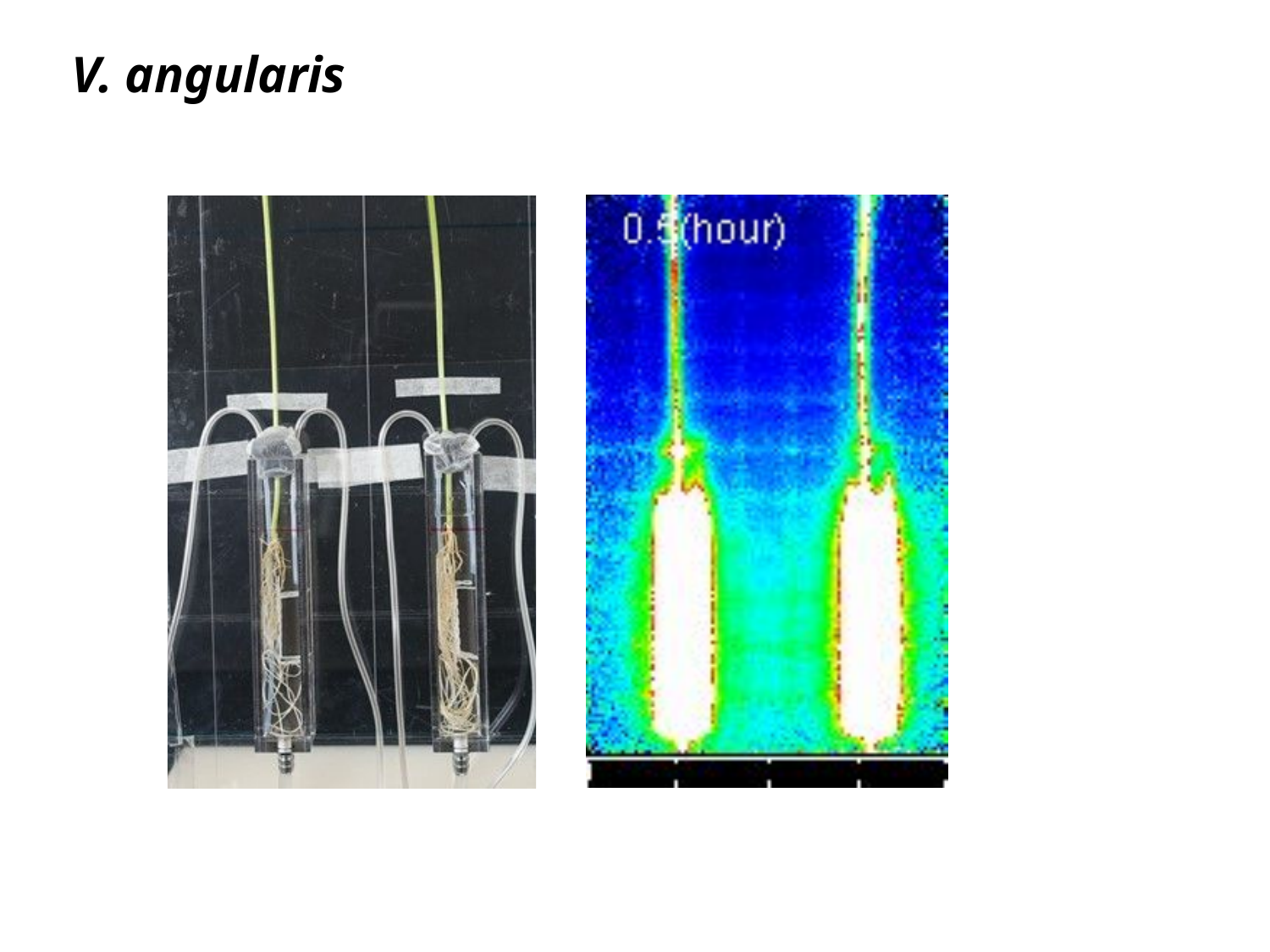

V. angularis

Supplement: Supplementary file 4 — Supporting information. [file PCE-48-3925-s001.pptx]
